# Methanol utilizers of the rhizosphere and phyllosphere of a common grass and forb host species

**DOI:** 10.1186/s40793-022-00428-y

**Published:** 2022-07-06

**Authors:** Saranya Kanukollu, Rainer Remus, Alexander Martin Rücker, Caroline Buchen-Tschiskale, Mathias Hoffmann, Steffen Kolb

**Affiliations:** 1 Microbial Biogeochemistry, RA1 Landscape Functioning, ZALF Leibniz Centre for Agricultural Landscape Research, Müncheberg, Germany; 2 Thaer Institute, Faculty of Life Sciences, Humboldt University of Berlin, Berlin, Germany; 3 Isotope Biogeochemistry and Gas Fluxes, RA1 Landscape Functioning, ZALF Leibniz Centre for Agricultural Landscape Research, Müncheberg, Germany; 4 Max Planck Institute for Biogeochemistry, Hans-Knöll-Straße 10, 07745 Jena, Germany; 5 Present Address: Johann Heinrich von Thünen-Institut, Institute of Climate-Smart Agriculture, Braunschweig, Germany

**Keywords:** Methylotrophs, Methanol, Rhizosphere, Metagenome-assembled genomes (MAGs), Radioactive labelling (^14^C-methanol), Potential methanol consumption rates

## Abstract

**Background:**

Managed grasslands are global sources of atmospheric methanol, which is one of the most abundant volatile organic compounds in the atmosphere and promotes oxidative capacity for tropospheric and stratospheric ozone depletion. The phyllosphere is a favoured habitat of plant-colonizing methanol-utilizing bacteria. These bacteria also occur in the rhizosphere, but their relevance for methanol consumption and ecosystem fluxes is unclear. Methanol utilizers of the plant-associated microbiota are key for the mitigation of methanol emission through consumption. However, information about grassland plant microbiota members, their biodiversity and metabolic traits, and thus key actors in the global methanol budget is largely lacking.

**Results:**

We investigated the methanol utilization and consumption potentials of two common plant species (*Festuca arundinacea* and *Taraxacum officinale*) in a temperate grassland. The selected grassland exhibited methanol formation. The detection of ^13^C derived from ^13^C-methanol in 16S rRNA of the plant microbiota by stable isotope probing (SIP) revealed distinct methanol utilizer communities in the phyllosphere, roots and rhizosphere but not between plant host species. The phyllosphere was colonized by members of *Gamma*- and *Betaproteobacteria*. In the rhizosphere, ^13^C-labelled Bacteria were affiliated with *Deltaproteobacteria*, *Gemmatimonadates,* and *Verrucomicrobiae.* Less-abundant ^13^C-labelled Bacteria were affiliated with well-known methylotrophs of *Alpha*-, *Gamma*-, and *Betaproteobacteria*. Additional metagenome analyses of both plants were consistent with the SIP results and revealed Bacteria with methanol dehydrogenases (e.g., *MxaF1* and *XoxF1-5*) of known but also unusual genera (i.e., *Methylomirabilis*, *Methylooceanibacter*, *Gemmatimonas*, *Verminephrobacter*). ^14^C-methanol tracing of alive plant material revealed divergent potential methanol consumption rates in both plant species but similarly high rates in the rhizosphere and phyllosphere.

**Conclusions:**

Our study revealed the rhizosphere as an overlooked hotspot for methanol consumption in temperate grasslands. We further identified unusual new but potentially relevant methanol utilizers besides well-known methylotrophs in the phyllosphere and rhizosphere. We did not observe a plant host-specific methanol utilizer community. Our results suggest that our approach using quantitative SIP and metagenomics may be useful in future field studies to link gross methanol consumption rates with the rhizosphere and phyllosphere microbiome.

**Supplementary Information:**

The online version contains supplementary material available at 10.1186/s40793-022-00428-y.

## Background

Managed grasslands are a global source of atmospheric methanol. Methanol is one of the most abundant and chemically reactive volatile organic compounds (VOCs; syn. volatiles) in the atmosphere [1]. Major sources of methanol is its release during plant growth and decay from the methoxy groups of plant structural compounds (1.5–45.7 µg per g dry weight h^−1^) and equals to 103 Tg year^−1^ [2–4]. Atmospheric methanol is a major contributor to tropospheric oxidant photochemistry, i.e., ozone formation [1, 5, 6]. Millet and coauthors [7] estimated the global methanol source as 242 Tg year^−1^. Despite a consensus regarding major sources and sinks (involving reaction with OH radicals), there are discrepancies between the annual global methanol production and release into the atmosphere by a factor of 3 to 4 [8, 9]. This suggests that plant-associated sink activities might be crucial in regulating net surface methanol emission rates through consumption, i.e., the amount of methanol formed and consumed and the amount of methanol that escapes into the atmosphere [10].

It is well known that methylotrophic bacteria use methanol as a sole and preferred source of carbon and energy [10, 11]. Methanol-utilizing methylotrophs are ubiquitous in terrestrial ecosystems and colonize plants [6, 10, 12–14]. Hence, they are an essential component of the plant holobiont and occur in the phyllo-, rhizo- and endospheres of plants. These methanol utilizers comprise representatives from *Alpha*-, *Beta*-, and *Gammaproteobacteria* and are further affiliated with *Verrucomicrobia*, *Actinobacteria*, *Firmicutes*, and *Flavobacteriia* [6, 15, 16]. A large proportion of the known methanol utilizers of plants colonize the phyllosphere [17, 18], and these members belong to the proteobacterial genera *Methylobacterium*, *Methylophilus*, *Methylibium*, and *Hyphomicrobium* [19–21]. A few prior studies on *Arabidopsis thaliana*, cereals, grasses, and pea plants revealed the presence of methanol dehydrogenase enzymes and methylotrophic bacteria of other proteobacterial genera, such as *Methylobacteraceae*, *Methylophilaceae*, *Methylocaldum*, and *Comamonadaceae,* in the rhizosphere microbiota [22–26]. However, information on the active methanol utilizer communities in the rhizosphere remains scarce.

**Fig. 1:**
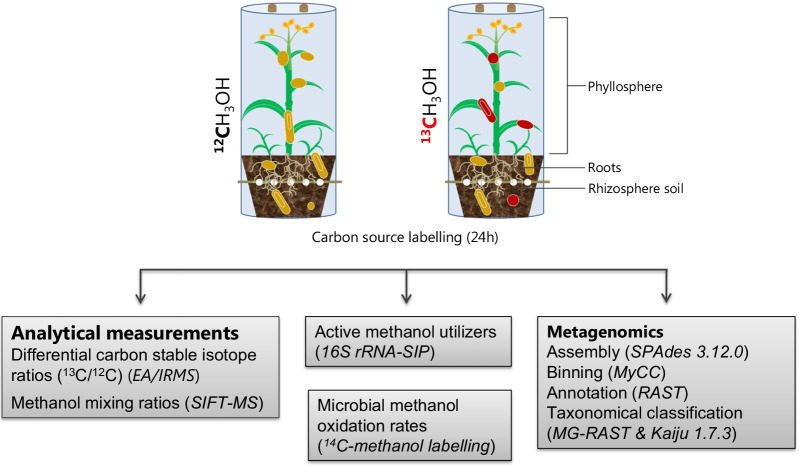
Workflow of the combined molecular approaches with RNA-SIP and metagenomics

Most of the prior investigations on plant-associated methylotrophs focused on targeted molecular approaches such as gene amplification with functional gene markers and 16S rRNA-based metabarcoding. To date, the commonly used functional gene markers have been target genes of the key enzyme methanol dehydrogenase (MDH), namely, *mxaF*, *xoxF*, and *mdh2*. Nonetheless, this approach has several limitations, such as being highly divergent, revealing little biodiversity and being limited to specific environments [6, 12, 16]. Thus, underestimation of the biodiversity of active methanol utilizers in plant-associated habitats by this method is very likely. Cultivation- and primer-independent techniques, such as metagenomics, may provide less biased insights into microbiota [27]. Our study combined RNA SIP and metagenomics to enable detailed identification of active plant-associated methanol utilizers and their metabolic capacities (Fig. 1).

The importance and roles of the microbiota in numerous biogeochemical processes, such as carbon (C) turnover and exchange with the atmosphere and regulation of greenhouse gases and further VOCs net surface fluxes are well known [28–31]. But previous studies rarely focused on the relationship between the microbiota and its relevance for net methanol surface emission rates from terrestrial ecosystems through consumption [32–34]. We aimed to identify key plant microbiota members involved in methanol consumption and to quantify the potential methanol consumption rate within the parts of those plant species that are important sinks. To characterise plant associated net methanol consumption, we employed two approaches, i.e., (i) a radioactive tracer method with ^14^C-methanol to reveal the methanol sinks within individuals of two grassland plant species and (ii) a field measurement to quantify net surface methanol fluxes using closed chambers and selected-ion flow-tube mass spectrometry (SIFT-MS) in a managed grassland with various plant species.

## Materials and methods

### Site description and growth of plants

The soil used to grow the grassland plants was taken from the Research Station Paulinenaue of the Leibniz Centre for Landscape research e.V. (ZALF) in the peatland complex “Haverländisches Luch” located in northeastern Germany (52° 41′ N, 12° 43′ E). The soil for growing grassland plants in subsequent experiments was collected from a permanent pasture situated in a shallow dip consisting of a weak moorshyfied fen soil type (0–17 cm horizon) [35]. This region is characterized by a continental climate with a mean annual air temperature of 9.2 °C and mean annual precipitation of 530 mm (1982–2012) [36]. The grassland area is regularly mown. The soil was stored overnight at 4 °C.

Two different grassland plants were investigated in this study: *Festuca arundinacea* and *Taraxucum officinale*. Plant seeds (Appels Wilde Samen GmbH, Germany) were placed into pots containing sampled soil from Paulinenaue. When seedlings reached the first growth state, plants were separated and watered weekly. Hoagland nutrient solution [37] (100 mL) was mixed in 1 L of distilled water once in a month. Plants were growing from March to August 2017 either outside at the ZALF Research Station in Müncheberg or inside a phytotron (Fitotron, Weiss Umwelttechnik GmbH, Germany) under the following conditions: 16 h of light at 20 °C and 8 h of dark at 14 °C, relative humidity of 70–80% and an illumination intensity of 80%. The outside conditions during the study period from March to August comprised a mean temperature of 18.2 °C, a mean precipitation of 278.2 L m^−2^ and a sunshine duration of 692.2 h.

### Airtight plant-growth chambers, labelling and sampling of plant material

Airtight plant-growth chambers (volume: 56.52 L; area: 706.5 cm^2^) were constructed by Reli Kunststoffe (Erkner, Germany). Acryl-glass material was used to construct the chambers, and airtight gas tubing made with butyl rubber, which is inert to methanol, was used to seal tubes. Gas-tight plant chambers were constructed with acryl glass (thickness: 5 mm). Butyl rubber stoppers with a three-way valve tube at the top and at the lower part of the chamber were used as ports for supplying ^13^C/^12^C CH_3_OH and extracting gas samples. A small ventilator in the lower part of the chamber ensured an even distribution of the gases in the chamber. The tightness test was performed by injecting CO_2_ into each chamber and measuring the concentration over time using an infrared gas analyser (LI-840, LICOR Biosciences, USA) (data not shown). Aluminium foil was wrapped around and above the chambers during incubation with ^13^C/^12^C CH_3_OH to reduce photosynthesis and microbial CO_2_ consumption during the labelling experiment (Additional file 1: Fig. S1).

Labelling experiments were conducted on intact plants (grown in a phytotron) in airtight plant growth chambers; working with intact plants can minimize the plant stress that leads to the release of methanol upon excision of the plant material. Six mature plants (> 12 weeks) of both plant species were used for the experiment. In addition to the control plants, four plants were placed in each plant growth chamber. Both plant compartments (phyllosphere and rhizosphere) were labelled separately with 1 mM ^12^C-CH_3_OH or ^13^C-CH_3_OH (Sigma-Aldrich, Merck KGaA, Darmstadt, Germany) (Additional file 1: Fig. S2,). For both plant species, the phyllosphere was separated from the rhizosphere using silicon (TACOSIL® 170, Thauer & Co. KG, Dresden, Germany) 12 h prior to the start of the experiment as described previously [38] to prevent the exchange of other gases.

Plant phyllosphere samples (leaves and stems), roots and rhizosphere soil were sampled immediately after each incubation period. Leaves were detached at the branches of the plant near the stem and cut into smaller pieces using scissors that were sterilized with 70% ethanol. Rhizosphere soil was mechanically separated from the roots by manual shaking. Smaller filaments of roots were still present in the rhizosphere soil. The remaining roots were subsequently cut into smaller pieces. The samples were immediately frozen using liquid N_2_ and stored at − 80 °C until further processing.

### Analysis of differential stable carbon isotope ratio (δ^13^C value)

From each plant, an aliquot of the leaves, roots and soil was sampled at the start of the experiment (0 h), after 8 h and at the end of the incubation period (after 24 h). Samples were dried for 24 h at 60 °C and finely ground using a vibrating disc mill (RS200, Retsch, Germany). Stable isotope ratios (^13^C/^12^C) were determined using an Elemental Analyzer (EA) Flash 2000 HT (Thermo Fisher Scientific, Bremen, Germany), which was coupled with a Delta V isotope ratio mass spectrometer (IRMS) via a ConFlo IV interface (Thermo Fisher Scientific, Bremen, Germany). Stable carbon isotope values (δ^13^C) were expressed as per mil (‰) relative to the international standard.δ^13^C values were normalized to the international scale Vienna Pee Dee Belemnite (VPDB) by analyses of the international standards USGS40 and USGS41 (L-glutamic acid) within the sequence [39]. The precision, defined as the standard deviation (± 1σ) of the laboratory control standard along the run, was smaller than ± 0.1‰

### Nucleic acid extraction and processing of RNA SIP gradient fractions

DNA was extracted from all plant material (phyllosphere, roots and rhizosphere soil) using the FastDNA™ Spin Kit for Soil (MP Bio Science Ltd., Derby, UK) following the manufacturer’s instructions. RNA extraction was performed using an RNeasy® Powerplant® Kit (Qiagen GmbH, Hilden, Germany). RNA-SIP of methanol-utilizing bacteria was carried out according to a previous study [40]**.** RNA gradient preparation and centrifugation were performed with a caesium tri-fluoroacetate (CsTFA) gradient as described elsewhere [41]. To establish the density gradient, the mixture was centrifuged for 72 h at 20 °C and 125,000×*g* (LE-70 Ultracentrifuge, Beckman Coulter GmbH, Krefeld, Germany). Fractions of 350 µl of the centrifuged gradients were separated, and their refractive index was determined at 20 °C with a digital refractometer (DRBO-45ND Müller Optronic; Erfurt, Germany). Finally, the RNA from the gradient fractions was precipitated with 400 µL of isopropanol and stored at − 80 °C until further processing. Reverse transcription of RNA to cDNA was performed with a Biozym cDNA Synthesis Kit (Biozym Scientific GmbH, Hessisch Oldendorf, Germany).

The primers 799F, labelled at the 5′-end with 6-carboxyfluorescein (6-FAM), and 1193r were used to amplify the 16S rRNA gene with a fragment length of 500 bp [42]. The PCR products were purified with an MSB Spin PCRapace Kit (Stratec Molecular GmbH, Germany) following the manufacturer’s instructions and subsequently checked via gel electrophoresis. Terminal restriction fragment length polymorphism (T-RFLP) was used to obtain bacterial community profiles as described elsewhere [43]. Data analysis of the T-RF profiles was performed using GeneMapper version 5.0 (Life Technologies, USA). Normalizing of T-RF frequencies was conducted [44]. To compare community structures treated with different parameters, the ordination technique of nonmetric multidimensional scaling (NMS) was applied using PCOrd version 6.08. The NMS was performed using Bray–Curtis dissimilarity, which does not overemphasize the variance in low-abundance T-RFs.

### 16S rRNA-based metabarcoding, data processing and identification of ^13^C-labelled methanol-consuming bacteria

All 60 labelled and fractionated samples and controls from the subsampling after 8 h and 24 h of incubation were investigated by amplicon sequencing using the primers 799F (5′-AACMGGATTAGATACCCKG-3′) and 115R (5′-AGGGTTGCGCTCGTTRC-3′). The hypervariable V3 region of the 16S rRNA gene was amplified. Pre-processing steps before sequencing by Illumina MiSeq were performed by LGC Genomics (Berlin, Germany). Data pre-processing and OTU building were performed with Mothur 1.35.1 [45]. Biodiversity index determination was performed with QIIME 1.9.0 [46]. Chimeras were removed with UCHIME (de novo and reference modes) using the most recent SILVA database (SSU119NR) as a reference dataset [47, 48]. Singletons (OTUs represented by only one sequence in the entire dataset) were removed. Consensus sequences were determined for each OTU at 3% genetic divergence using USEARCH and classified by BLAST alignment against the SILVA SSURef 119 NR database [49]. Sequences were classified with respect to the SILVA taxonomy of the best hit. Rarefaction curves and Shannon diversity indices [50] were calculated as previously described [51]. In addition, the maximal number of OTUs (n_max_) was estimated for each sample using the Michaelis–Menten function fit. The OTUs were analysed for confirmation of ^13^C-labelled microbe-specific selection criteria as described previously [52, 53]. A few modifications were made to those criteria due to the labile nature of RNA, i.e., (1) the relative abundance of a specific taxon in the ^13^C treatment’s heavy fraction should be higher than that in same fraction of the ^12^C-control treatment; (2) the relative abundance of a specific OTU in the heavy fraction should be higher than that in the light fraction of the gradient of the 13C treatment by a factor K = 2, due to the substrate-based stable isotope approach and short incubation periods; and (3) the relative abundance of a specific OTU in the heavy fraction of the ^13^C treatment should be larger than or equal to 0.05%.

### Metagenomes from both plant species including bioinformatics analyses

Metagenome sequencing was performed for two DNA samples that were pooled together in equimolar amounts from plant material (phyllosphere, roots, rhizosphere soil) of both plant species incubated with ^13^C-CH_3_OH for 8 h. Sequencing was performed on the Illumina NextSeq platform, and raw read data were pre-processed and trimmed by a commercial service (LGC Genomics GmbH, Berlin). Taxonomical analysis of the processed and trimmed reads was performed with Kaiju version 1.7.3 [54]. Processed reads were assembled with SPAdes 3.12.0, which includes the metaSPAdes pipeline (Nurk & Bankevich et al., 2013) with default options. QUAST v4.0 was used to check the assembly statistics for both metagenomes (Table 1). The rarefaction curves of both metagenomes achieved sufficient coverage (Additional file 1: Fig. S3).

**Table 1:** QUAST assembly statistics of metagenomes from plants incubated with ^13^C-CH_3_OH for 8 h (phyllosphere, roots and rhizosphere soil)

	*Festuca arundinacea*	*Taraxacum officinale*
# contigs (≥ 0 bp)	10,475,105	8,330,240
# contigs (≥ 1000 bp)	96,819	46,165
Total length (≥ 0 bp)	3,136,960,356	2,322,513,829
Total length (≥ 1000 bp)	180,390,757	82,360,988
Total length	512,099,929	264,370,104
GC (%)	62.78	60.38
N50	774	734
N75	594	581
L50	183,904	105,423
L75	375,536	207,866

Metagenomes were obtained by shotgun sequencing of pooled ^13^C-labelled DNA from both the phyllosphere and rhizosphere of the plants incubated with ^13^C-CH_3_OH for 8 h.

Assembled contigs were again taxonomically and functionally classified using the MG-RAST server [55]. KEGG pathways for methanol assimilation were also obtained from MG-RAST. Assembled sequences were binned using MYCC [56] with 4mer and a minimum contig length of 1000 bp. The coverage profiles were produced through MYCC and BAM files using Bowtie 2 and MetaBAT to produce a depth file. The quality of the bins was estimated using CheckM v1.1.2 [57]. Bins with a reported completeness > 70% and contamination < 10% were selected as metagenome-assembled genomes (MAGs) for further analysis. MAGs were annotated with RAST-tk [58], and the closest taxon was used as the initial taxonomic classification. Desired protein sequences (MDH genes) from the annotated bins were downloaded from RAST as ‘.faa’ files. Further evaluation of MDH genes (*mxaF*, PQQ-dependent gene, *xoxF*1-5) in MAGs was performed using BLAST [59] and RAST [60]. They were screened exclusively for the functional marker genes involved in methanol utilization. Phylogenetic trees were reconstructed with MEGA7 using the maximum likelihood method from MSAs and the JTT + G + I evolution model with n = 500 bootstrap replications.

### Accession numbers of nucleotide data

Read data of the two metagenomes were published in the National Center for Biotechnology Information (NCBI) database under BioProject number PRJNA715626. Raw data amplicons of all 60 samples were deposited in NCBI sequence short-read archive under the same BioProject (PRJNA715626) with accession numbers SRA14001742 to SRA14001801.

### Radioactive labelling with ^14^C_1_-methanol to determine potential methanol consumption rates

To quantify potential methanol turnover rates in the phyllosphere, roots and rhizosphere soil of the investigated plants, freshly excised plant material (leaves, roots, or rhizosphere soil) was incubated with ^14^C-CH_3_OH for 3.5 h at 20 °C (performed in a climate chamber). Biological oxidation of ^14^C-CH_3_OH releases ^14^C-CO_2_. The released CO_2_ was trapped in 12 mL of 1 M NaOH solution. Vapourized ^14^C-CH_3_OH was trapped in 12 mL of water (Additional file 1: Fig. S4). A new and safe trapping system was designed (Additional file 1: Supplementary Fig. S5). The plant material (leaves, roots, rhizosphere soil) was incubated with 631 kBq of ^14^C-CH_3_OH for 3.5 h in 4 replicate glass bottles (SCHOTT DURAN, 100 mL), each with a sterile inlet and outlet for gases placed in a climate chamber on a shaker. The inlet of the glass bottle was connected to test tubes with water to maintain humidity. The outlet was connected to both a CO_2_ trap (NaOH solution) and a methanol trap (water). The water to trap vapourized ^14^C-CH_3_OH was maintained at 3 °C using a cryostat. Thus, evaporation of condensed methanol in water was prevented. For all the traps, test tubes (three of them, always connected in parallel) with 12 mL of water or NaOH were used. At the rear end, after the ^14^C-methanol trap, a mass flow controller with a constant gas flow (15 mL min^−1^) and a pump were installed. During the experiment, test tubes with 1 M NaOH solution (CO_2_ traps) were collected every 30 min, while methanol traps with cold water were collected only once at the end of the experiment.

The activity of ^14^C in all the traps was determined by a TriCarb 2900 TR liquid scintillation counter (PerkinElmer). The scintillation mixture (15 mL) was prepared with 12 mL of UltimaGold (PerkinElmer) and either 3 mL of pooled NaOH solution or water from both the CO_2_ and methanol traps. Linear regression analyses were used to determine the slope of methanol turnover for each incubation setup, where the mean R^2^ of all 24 incubation experiments was 0.986–0.017 and 1. Then, the slope values and dry biomass of excised plant material (leaves, roots, and rhizosphere soil) from both plant species (*F. arundinacea* and *T. officinale*) were used to determine the methanol turnover rate (nmol g dry wt^−1^ h^−1^). The dry biomasses of leaves, roots, and rhizosphere soil were 50, 32 and 28 mg, respectively. The efficiency of the labelling method was tested by measuring the total content of CO_2_ collected in NaOH using 0.5 M BaCl_2_. Precipitated CO_2_ (BaCO_3_) was washed on a membrane filter with ultrapure water and dried at 104 °C. Then, the membrane filters with BaCO_3_ were combusted at 1350 °C (multi EA 4000, Analytik Jena, Germany) with a continuous flow of oxygen, and the resulting CO_2_ was trapped in 7 mL CarboSorb E (PerkinElmer). Subsequently, 3 mL of the CarboSorb E sample was mixed with 12 mL of Permafluor E + (PerkinElmer), and then, the activity of ^14^C was determined by using a liquid scintillation counter. The total activity of all BaCO_3_ precipitates determined here was 3.3% more than the expected value resulting from the ^14^C activities of the NaOH samples. This result indicates a minor methodological error. However, the accumulation of volatile methanol in the CO_2_ traps can be excluded. In addition, the linear regression analysis of the ^14^C activities of the NaOH and BaCO_3_ samples showed an R^2^ of 0.945.

## Results and discussion

Previous investigations on methanol utilizers have mostly focused on forest soils [6, 12]. Fewer studies on plant-associated methanol utilizers have provided valuable insights and highlighted their importance in global methanol emissions [19, 22, 24]. Recently, Macey and coauthors revealed the importance of methanol utilizers in bulk and plant-associated soils using a combined approach with molecular probes, DNA SIP and metagenomics [52]. Nevertheless, by separating the rhizosphere soil from the plant, they provided the first evidence that the rhizosphere has methanol consumption activity. However, the study only analysed rhizosphere soil after destructive sampling. Our study provides detailed information about the role of methanol utilizers in intact plant methanol consumption rates and active methanol-incorporating bacteria, and we were still able to experimentally separate the phyllosphere and rhizosphere without harming the plants.

### Active bacterial methanol utilizers of the phyllosphere and rhizosphere of both plant species

**Fig. 2:**
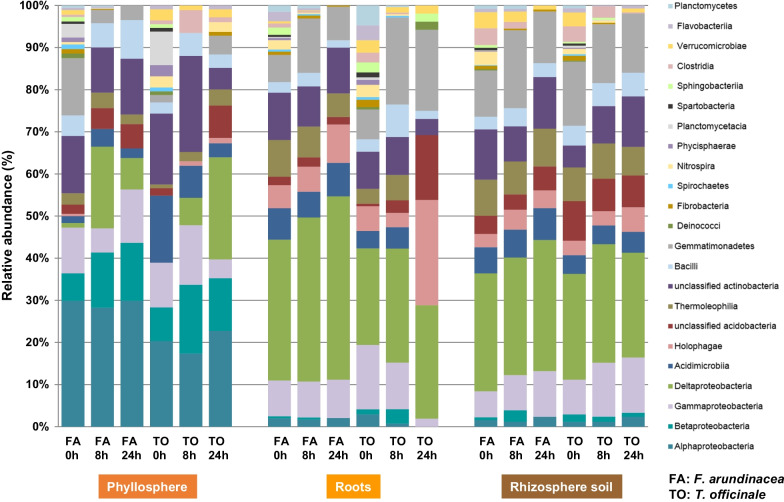
Divergent bacterial ^13^C-labelled 16S rRNA OTU profiles between the plant compartments. The relative abundance of ^13^C-labelled bacterial profiles is shown at the class level for both the plant species (*F. arundinacea* and *T. officinale*) between different plant compartments (phyllosphere, roots and rhizosphere soil). Both plants were incubated with 1 mM ^13^C-CH_3_OH for 0 h, 8 h and 24 h

It is well known that RNA has higher sensitivity and exhibits more rapid metabolic turnover than DNA [61]. Thus, using RNA SIP instead of DNA SIP to identify active methanol utilizers have an added advantage in our study; we avoided unnecessarily long incubation periods and hence minimized potential stress for the plants caused by labelling in closed chambers. RNA SIP revealed distinct methanol utilizer communities in the phyllosphere, rhizosphere and roots of both plant species (Fig. 2). Both plant species shared higher similarities in methanol utilizer communities, while larger differences in alpha biodiversity (Chao1) were mainly identified at the species level among all plant compartments (Additional file 1: Fig. S6). NMS ordination based on 16S rRNA gene TRFLP profiles of the active bacteria from both the plant species also confirmed the RNA SIP plant pattern (Additional file 1: Fig. S7). Here, a separated clustering of the samples from the phyllosphere was observed, while roots and the rhizosphere soil samples clustered together and were dispersed. This impact was in consistent with an earlier study in temperate grasslands [62].

The predominantly detected taxa in both the phyllosphere and rhizosphere were members of *Alphaproteobacteria*, *Betaproteobacteria*, *Gammaproteobacteria,* and *Actinobacteria* (Fig. 2). The presence of *Actinobacteria* in all plant compartments was evident and consistent with previous studies analysing plant growth-promoting bacteria [63, 64]. The phyllosphere microbiota was mostly dominated by the members of *Comamonadaceae*, *Methylophilaceae* [62], *Methylococcaceae*, *Sphingomonadaceae* and *Pseudomonadaceae* (Fig. 2). Members of *Sphingomonadaceae* and *Pseudomonadaceae* are known to be the most abundant representatives in leaf epiphytic microbiota and can degrade various plant-derived carbon compounds [65, 66]. *Deltaproteobacteria*, *Gemmatimonadetes, Holophagae, and Verrucomicrobiae* were the predominant ^13^C-labelled bacterial classes in the roots and rhizosphere (Fig. 2). Greater species richness was observed in both roots and rhizosphere soil than in the phyllosphere. The predominant ^13^C-labelled bacterial taxa (≥ 1% relative abundance) were *Methylophilaceae*, *Hyphomicrobiaceae*, *Comamonadaceae, Sphingomonadaceae, Solirubrobacteraceae*, *Rhizobiaceae*, *Methylobacteriaceae* and *Xanthomonadaceae* (Additional file 1: Fig. S8). Irrespective of plant species and compartments, the methanol utilizer communities overlapped in terms of composition between the phyllosphere and rhizosphere. This proves their significance in methanol consumption fluxes in both investigated plant hosts. The presence of *Methylophilaceae* in both the phyllosphere and rhizosphere was consistent with previous studies on bacteria associated with plants [24, 52]. *Methylophilaceae* is also known as the only bacterial family that harbours the *xoxF4* gene among all known methanol utilizers [52, 67, 68]. Members of *Comamonadaceae* are known to contain *xoxF* genes, and their capability to degrade methanol has been previously reported [69, 70]. Species of *Xanthomonadaceae* are known as plant pathogens and the most abundant root exudate utilizers in the rhizosphere [63]. However, the presence of the *xoxF*1 methanol dehydrogenase gene suggests methylotrophic potential [71]. This knowledge supports our conclusion that the ^13^C-labelled taxa were indeed methylotrophs.

### The role of the detected *Gemmatimonadetes* and *Acidobacteria*

**Fig. 3:**
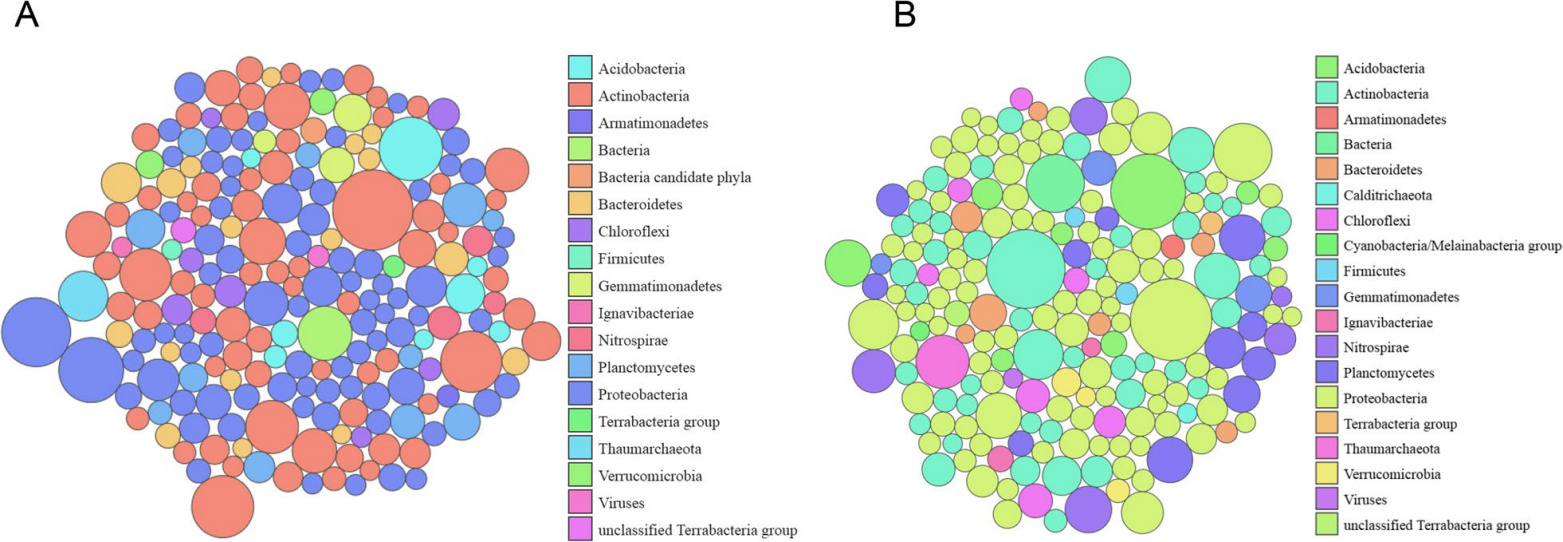
Metagenome-derived taxonomic classification of plants incubated with.^13^C-CH_3_OH (phyllosphere, roots and rhizosphere soil). Quality trimmed reads from shotgun sequencing were subjected. The bubble plots show the relative abundances of taxa (phylum level) comprising at least 0.1% of classified reads. The size of each bubble is scaled logarithmically to depict the abundance of each taxon relative to its maximum abundance (largest bubble size). The size of the circle is scaled logarithmically to represent the number of sequences assigned directly to the taxon. **A** Microbial community composition of the *F. arundinacea* metagenome where all the plant materials were pooled together in equimolar amounts. **B** Microbial community composition of the *T. officinale* metagenome where all the plant materials were pooled together in equimolar amounts

The presence of *Gemmatimonadetes* in both metagenomes and in the active methanol community as revealed by RNA SIP is striking and suggests that these species are methanol utilizers (Figs. 2, 3). Despite their ubiquitous presence and high abundance (ca. 2%) in many rhizosphere and soil studies, they are frequently ignored due to a lack of cultivable isolates [72]. Representatives from this phylum are often involved in nitrogen and sulphur cycles, but recent studies on their genomes revealed the presence of MDH genes, and thus, can be considered as methylotrophs [73]. We also detected members of the phylum *Acidobacteria* (*Holophagae*). This phylum is also lacks cultivable isolates and was found to be abundant in a study on methanol-utilizing bacteria in a forest soil [74]. Recently, a member of *Acidobacteria* (*Solibacter*) was described as a methylotroph. It possess the *xoxF3* gene. Thus, our study proved that these phyla that have been long overlooked in regard to methanol utilization are relevant in common grassland plant hosts for methanol turnover.

### Identification of methanol-assimilating Bacteria associated with both plant species by metagenome analysis

Metagenomes of both plant species revealed similar biodiversities and the presence of unusual methanol utilizers. Taxonomic analyses of the processed reads and the assembled contigs of both metagenomes were dominated by the domain Bacteria, particularly by members of *Proteobacteria* (45%), *Actinobacteria* (26%), *Bacteroidetes* (7%), *Verrucomicrobia* (3%) and *Firmicutes* (5%) (Fig. 3, Additional file 1: Fig. S9). The predominance of these members was expected since they have often been identified in various studies on methanol-degrading microbes. However, based on average frequencies in both metagenomes, a few phyla were abundant in our study and have gone unnoticed thus far, such as *Deltaproteobacteria* (6%), *Plantomycetes* (3.5%) *Acidobacteria* (3%), and *Gemmatimonadetes* (1%). The highly abundant genera and families in both metagenomes were consistent with previous studies on methanol utilizers in plants and soils [14–16, 22, 52] and with the active members detected by RNA-SIP (Fig. 2). For members of all these bacterial genera (i.e., *Bradyrhizobium*, *Hyphomicrobium*, *Methylophilus*, *Mesorhizobium*, *Flavobacterium, Gemmatimonas, and Verminephrobacter)*, utilization of methanol is likely or has been indicated. *Bradyrhizobium* strains exhibit significant MDH activity and express *xoxF* in the presence of La^3+^ [75]. Members of *Hyphomicrobium* are frequently detected and isolated due to their wide distribution and their ability to use methanol as a carbon source, even at very low concentrations [76, 77]. The detection of *Flavobacterium* species in methanol-based studies is not unusual, but there is a knowledge gap regarding their methanol utilization capacity, necessitating further investigations on their growth substrate spectrum [78, 79].

The significantly abundant genes in both metagenomes were assigned to functional categories (KOs, COGs and subsystems). The distribution of the functional categories was remarkably similar (Additional file 1: Fig. S10). KEGG analysis of the functional categories revealed the presence and best hits of the whole methanol assimilation pathway in both metagenomes (Additional file 1: Fig. S11).

**Fig. 4:**
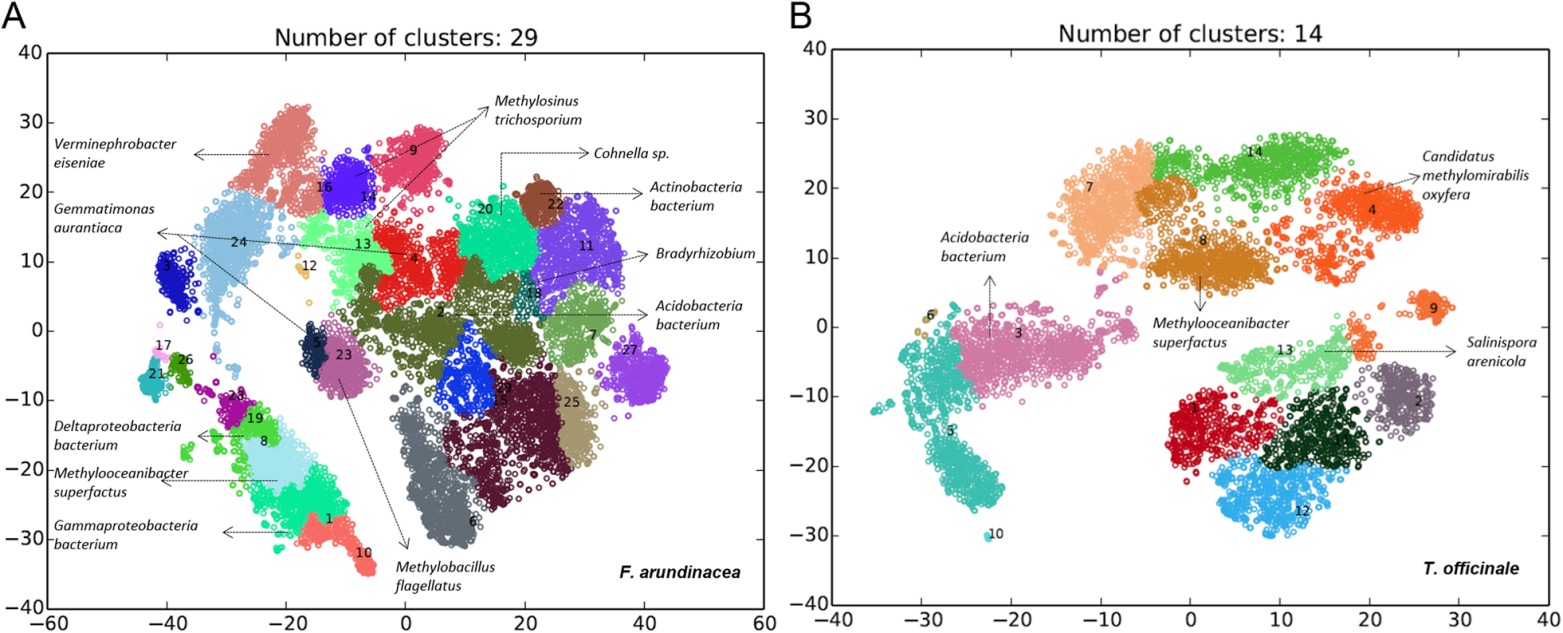
Metagenome-assembled genomes (MAGs) of ^13^C-CH_3_OH-treated plants (phyllosphere, roots and rhizosphere soil) harbouring MDH (PQQ, *mxaF*, *xoxf*1-5) genes. Processed reads were assembled with SPAdes 3.12.0, and then, the contigs were binned using MYCC. The quality of the bins was estimated using CheckM v1.1.2. Bins with a reported completeness > 70% and contamination < 10% were selected as MAGs and annotated with RAST-tk [67], and the closest taxon was used as the initial taxonomic classification. **A** MAGs of whole *F. arundinacea* plants incubated with ^13^C-CH_3_OH for 8 h revealed 29 annotated bins that harboured MDH genes. **B** MAGs of whole *T. officinale* plants incubated with.^13^C-CH_3_OH for 8 h revealed 14 annotated bins that harboured MDH genes

The complexity of metagenomic datasets and their processing can lead to a high level of genome fragmentation and heterogeneity, which might shift the microbe distribution patterns and can imbricate the microbiota compositions [80, 81]. These technical challenges can be overcome by binning, as these approaches often use abundance information from scaffolds or contigs. Binning of the contigs from MAGs of both plant species revealed 29 and 14 annotated bins (Fig. 4). Annotated bins identified as Bacteria were shortlisted. All genome bins were screened for PQQ-dependent MDH gene markers, such as *mxaF* or *xoxF* (1–5) (Table 2). Both plant species were dominated by typical representatives from *Alpha*-, *Beta*-, and *Gammaproteobacteria* and *Actinobacteria* and a few unexpected members affiliated with *Deltaproteobacteria, Acidobacteria, Gemmatimonadetes* and *Bacilli.* The genera of methanol utilizers detected in metagenomes from both MAGs were *Methylobacillus*, *Methylosinus*, *Methylomirabilis*, *Methylooceanibacter*, *Gemmatimonas* and *Verminephrobacter* (Fig. 4). A few detected members of *Acidobacteria*, *Gemmatimonadetes* and *Bacilli* (e.g., *Gemmatimonas* and *Verminephrobacter*) have never been detected previously. However, these taxa have been observed in many soil- and plant-associated habitat-based studies on methylotrophs. Therefore, we aimed to provide metabolic insights into these methanol utilizers. Only a few recent 16S rRNA-based, metagenome- and proteogenome-targeted studies have suggested the presence of methylotrophy in low-abundant phyla (e.g., *Acidobacteria*, *Gemmatimonadetes* and *Firmicutes*), thus suggesting their role in terrestrial methanol consumption [26, 52, 82]. Interestingly, in a recent proteome study, a PQQ-dependent MDH from *Gemmatimonadetes* was detected as the most abundant protein [26]. The limitations of such multi-omic studies on functional capacities have led to underestimation of the common and relatively low abundant members in soil- and plant-associated microbiota.

**Table 2:** Methanol dehydrogenase (MDH) genes in methanol utilizers from MAGs

Species level	Class level	MDH gene	Percent identity	Method
*Methylosinus trichosporium*	*Alphaproteobacteria*	xoxF	98%	Phylogenetic analysis (maximum likelihood)
*Cohnella *sp.	*Bacilli*	PQQ-dependent	95%	RAST/protein BLAST
*Uncultured Actinobacteria *sp.	*Actinobacteria*	PQQ-dependent	98%	RAST/protein BLAST
*Uncultured Acidobacteria *sp.	*Acidobacteria*	PQQ-dependent	94%	RAST/protein BLAST
*Methylobacillus flagellatus*	*Betaproteobacteria*	mxaF	100%	Phylogenetic analysis (maximum likelihood)
*Uncultured Gammaproteobacteria *sp.	*Gammaproteobacteria*	PQQ-dependent	95%	RAST/protein BLAST
*Methylooceanibacter Superfactus*	*Alphaproteobacteria*	xoxF1	92%	Phylogenetic analysis (maximum likelihood)
*Uncultured Deltaproteobacteria *sp.	*Deltaproteobacteria*	PQQ-dependent (MoxR gene)	96%	RAST/protein BLAST
*Gemmatimonas aurantiaca*	*Gemmatimonadetes*	PQQ-dependent	98%	RAST/protein BLAST
*Verminephrobacter eiseniae*	*Betaproteobacteria*	xoxF5	68%	Phylogenetic analysis (maximum likelihood)
*Candidatus* Methylomirabilis oxyfera	*Candidate division NC10*	xoxF1	90%	Phylogenetic analysis (maximum likelihood)
*Salinispora arenicola*	*Actinobacteria*	PQQ-dependent	95%	RAST/protein BLAST

RAST-annotated bins are listed at the species and class levels. They were screened for the presence of MDH genes using three different methods, namely, RAST, protein BLAST (NCBI) and phylogenetic analysis (MEGA7). Percent identity with respect to MDH genes was also obtained using the abovementioned methods

**Fig. 5:**
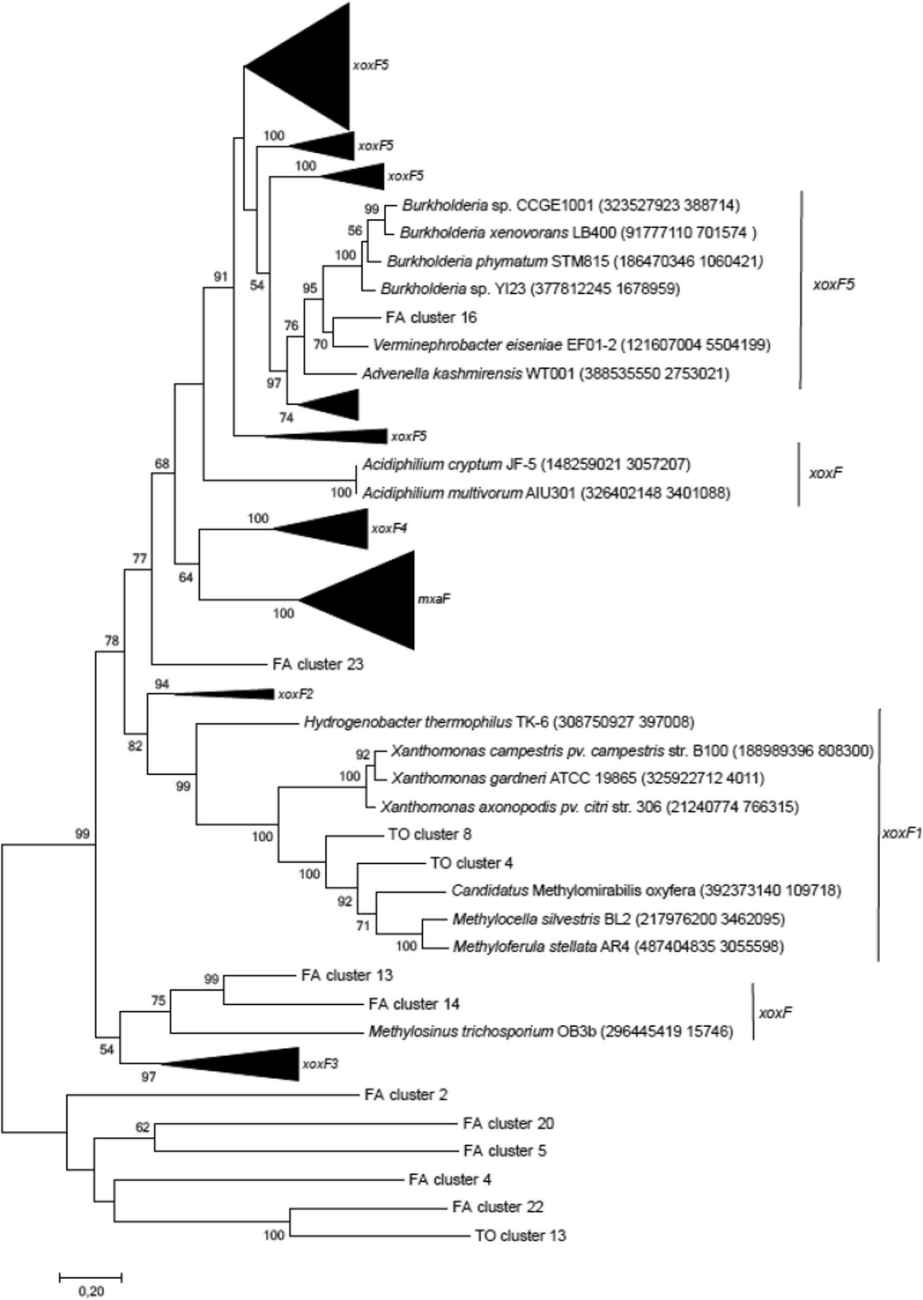
Molecular phylogenetic analysis of selected MAGs with MDH genes retrieved from both *F. arundinacea and T. officinale* plants incubated with ^13^C-CH_3_OH for 8 h. Phylogenetic analyses were conducted in MEGA7 [87] using the maximum likelihood method based on the JTT matrix-based model. The numbers at the branch nodes refer to bootstrap values. Only bootstrap values ≥ 50% (based on 500 replicates) are labelled at branch points. There were a total of 201 amino acid residues in the final dataset

Phylogenetic analysis of the binned contigs from both plants showed clear branching of metagenomic bins within the *xoxF* (1–5) gene-harbouring methylotroph datasets (Fig. 5). *T. officinale* annotated metagenome bins (clusters 4 and 8) were closely affiliated with *Candidatus* Methylomirabilis oxyfera. *F. arundinacea* annotated metagenome bins (clusters 13 and 14) were closely affiliated with *Methylosinus trichosporium* OB3b. Both *Candidatus* Methylomirabilis oxyfera and *Methylosinus trichosporium* are well known for their aerobic methane oxidation metabolism and possess different MDH genes (i.e., *MxaF1*, *XoxF1*, *XoxF2*, *XoxF3*, and *XoxF5*) [83, 84]. The close branching of both the *F. arundinacea* and *T. officinale* metagenome bins to *Candidatus* Methylomirabilis oxyfera and *Methylosinus trichosporium* suggests that the plant-associated methanol utilizers are adaptable and can easily switch their lifestyles with an available carbon source. Approximately 9 metagenome bins had distinct clustering. Taxonomic screening of those bins revealed their affiliation with the phyla *Actinobacteria* (clusters 22 and 20 of *F. arundinacea* annotated metagenome bins and cluster 13 of *T. officinale* annotated metagenome bins) and *Acidobacteria* (cluster 2 of *F. arundinacea* annotated metagenome bins) and the species *Gemmatimonas aurantiaca* (clusters 4 and 5 of *F. arundinacea* annotated metagenome bins). All these bins carried the MDH subunit gene *mxaF* (Table 2). Interestingly, one of the *F. arundinacea* annotated metagenome bins (cluster 16) grouped with *Verminephrobacter eiseniae EF01-2*. This species was recently shown to have methanol oxidation ability and to harbour MDH genes, specifically *xoxF* [85]. Thus, our study provided for the first time the relevance of these low-abundance and often overlooked bacterial taxa for a potential methanol consumption in common and temperate grassland soil systems.

### ^13^C labelling with intact plants but with a phyllosphere- and rhizosphere-separated labelling procedure allowed the affiliation of detected methanol utilizers within these compartments

Previous studies on plant-associated methylotrophic bacteria were conducted with cut-off aboveground plant material. However, it is well known that the VOC emissions of plants may change due to physical stresses such as tissue cutting. Leaf emissions are one of the major sources of methanol. Labelling studies are often conducted on cut-off leaves, and the carbon source provided is most rapidly preferred by most abundant epiphytes over endophytes, leading to other crucial methanol utilizers being overlooked. Due to their low relative abundances, the above described ‘unusual’ methanol utilizers have never been detected and identified. We applied, for the first time, a ^13^C-labelling approach in a plant microbial interaction study to separately label the phyllosphere and rhizosphere compartments while leaving the plants intact. Hence, we could exclude the exchange of the labelled compounds between the plant compartments. We also ensured that sufficient ^13^C labelling occurred by investigating ^13^C incorporation within 8 h and 24 h of incubation with ^13^C-CH_3_OH by subjecting the plant material (leaves, roots and rhizosphere soil) to further analyses or by examining their associated microbial communities using EA/IRMS (Additional file 1: Fig. S12). δ^13^C values of unlabelled leaves and roots of *F. arundinacea* were − 28.8 to − 26.8 ‰ and with − 30.7 to − 27.0 ‰.slightly heavier of *T. officinale* (Additional file 2: Table S1). Nevertheless, δ^13^C values were within the range for measured C_3_ plants under natural conditions [86]. The δ^13^C values of ^13^C-methanol-labelled leaves and root samples showed an almost threefold increase in ^13^C between the 8 h and 24 h samples compared to unlabelled samples (Additional file 1: Fig. S12). The enrichment in δ^13^C values in both species compared to the unlabelled samples probably indicates a higher abundance of methanol utilizers on leaf surfaces. This effect was not as evident in the rhizosphere soil samples. However, given our amplicon- and metagenome-based observations, we conclude that ^13^C incorporation from supplemented methanol had occurred.

### The rhizosphere microbiota is an important plant host-associated methanol sink in grasslands

We used radioactive isotope turnover measurement with ^14^C-methanol as a tracer to reveal potential methanol consumption rates in the investigated grassland species in all plant compartments. We used newly developed water traps to determine radioactivity loss through evaporated ^14^C-methanol. The water was cooled at 3 °C for maximal trapping of ^14^C-methanol. The efficacy was 97.5% with only 0.23% methanol in CO_2_ traps. Thus, we were able to quantify the potentially higher recovery rates for CO_2_ formation, which we used to calculate potential methanol consumption rates.

**Fig. 6:**
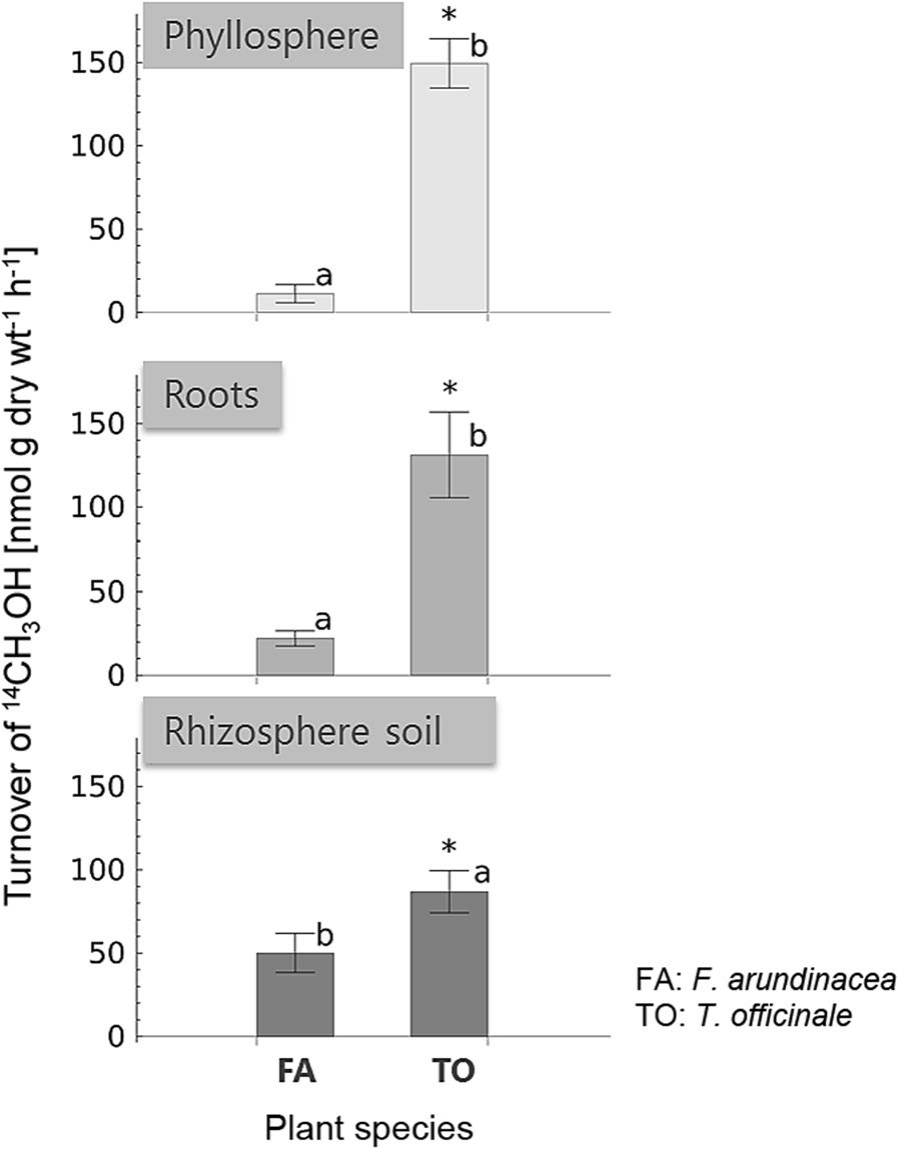
Potential methanol consumption rates in the phyllosphere, roots, and rhizosphere soil from both plant species. Plant material from both the plant species (*F. arundinacea* and *T. officinale*) was incubated with 631 kBq of ^14^C-CH_3_OH for 3.5 h. ^14^C activity was determined in CO_2_ traps with 1 M NaOH solution by using a liquid scintillation counter (TriCarb 2900 TR, PerkinElmer). Here *: show significant differences in the pairwise t test (p = 0.05) and different letters show significant differences in the one-way ANOVA with Tukey test (*p* = 0.05) for each plant species. Error bars are standard deviations (SD).

The methanol consumption rates were dependent on the plant species. *T. officinale* samples had higher rates than *F. arundinacea* samples (Fig. 6). The phyllosphere of *T. officinale* exhibited the highest methanol consumption rates (149 ± 15 nmol g dry wt^−1^ h^−1^). Roots of *T. officinale* had higher methanol consumption rates (131 ± 26 nmol g dry wt^−1^ h^−1^) than the rhizosphere soil (87 ± 12 nmol g dry wt^−1^ h^−1^), while *F. arundinacea* revealed the opposite trend. The rhizospheres (roots and rhizosphere soil) of both plant species showed similarly high methanol consumption rates as the respective phyllosphere compartments. ANOVA (two-way and one-way), *T* test and Welch’s test revealed a significant difference between both the plant species and plant materials (α = 5%). Thus, our study proved that the rhizosphere of the two common grassland plant host species is a highly active and therefore relevant methanol sink in such ecosystems.

**Fig. 7:**
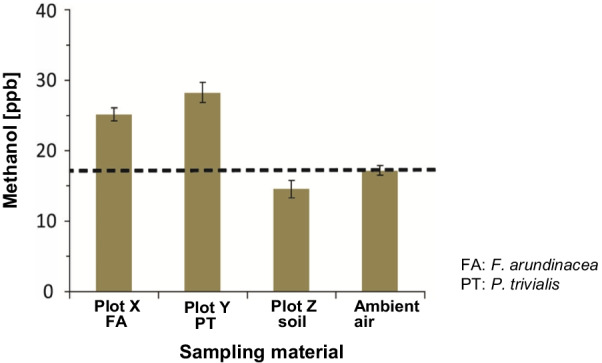
Methanol mixing ratios from experimental plots and ambient air after chamber closure. Samples from the experimental plots with *F. arundinacea*, *Poa trivialis*, and soil collected after only 15 min, 30 min, 60 min and 120 min of chamber closure were analysed by SIFT-MS. For comparison, methanol mixing ratios from the “soil only” plot are shown. The dashed line, background levels of methanol in ambient air. Error bars, technical replicates (n = 3)

Spot check quantification of methanol formation using closed chambers and SIFT-MS in a managed grassland provided insights into sources and sinks within the plant holobiont (Additional file 1: Fig. S13; Additional file 3: Supplementary information). Methanol mixing ratios and their rate change in the air from three experimental plots with two different plant species (approx. 26.5 ± 1.2 ppb) showed higher methanol concentrations than the plot without plant biomass (17.1 ± 0.7 ppb) (Fig. 7, Additional file 4: Table S2). Therefore, we confirmed that both above- and belowground plant parts are net methanol emitters.

## Conclusions

Our study revealed the rhizosphere of temperate grassland plant species as an overlooked local methanol sink and revealed new bacterial taxa that together with known ones represent the plant host-associated methanol sink. This finding led us to re-evaluate the canonical concept of members of the family *Methylobacteriaceae* being the key sink of methanol in plant species. The rhizosphere of both plant species was identified as a major sink for methanol in terrestrial ecosystems. To our knowledge, there has been no previous study that quantified this sink activity in the plant rhizosphere by direct measurements. Our study confirms a long-held assumption that the rhizosphere is one of the hotspots for methanol consumption in grasslands. Eventually, this finding implies that the observed net surface methanol emissions and consumption from grasslands and their responses to land use and climate change can be understood only if the belowground microbiota and its activity are considered.

## Supplementary Information

**Additional file 1.** Supplementary figures.

**Additional file 2**. Supplementary Table 1.

**Additional file 3.** Supplementary information on the evaluation of methanol formation in a managed grassland.

**Additional file 4.** : Supplementary Table 2.

## Data Availability

Read data of the two metagenomes have been submitted to the National Center for Biotechnology Information (NCBI) database under BioProject number PRJNA715626. Raw read data of all 60 samples were deposited in the NCBI sequence short-read archive under the same BioProject (PRJNA715626) with accession numbers SRA14001742 to SRA14001801.
