# Supplementary material for: Methanol utilizers of the rhizosphere and phyllosphere of a common grass and forb host species
Source: Environ Microbiome. 2022 Jul 6;17:35. doi: 10.1186/s40793-022-00428-y (PMC9258066; doi:10.1186/s40793-022-00428-y)
Supplement: Supplementary file 1 — Additional file 1. Supplementary figures. [file 40793_2022_428_MOESM1_ESM.pptx]

## Slide 1
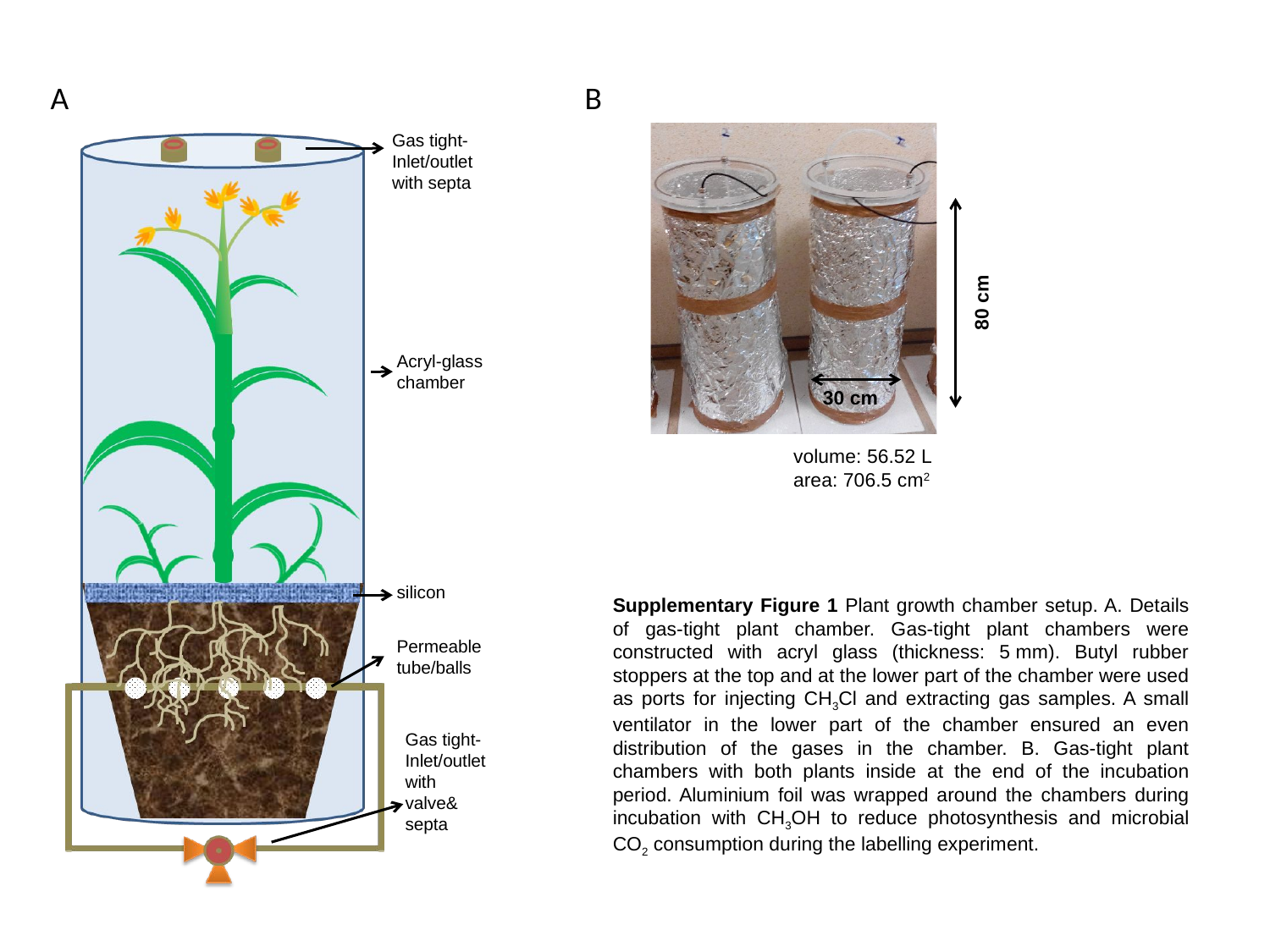

A
B
Gas tight-Inlet/outlet with septa
Acryl-glass
chamber
silicon
Permeable tube/balls
Gas tight-Inlet/outlet with valve& septa
80 cm
30 cm
volume: 56.52 L
area: 706.5 cm2
Supplementary Figure 1 Plant growth chamber setup. A. Details of gas-tight plant chamber. Gas-tight plant chambers were constructed with acryl glass (thickness: 5 mm). Butyl rubber stoppers at the top and at the lower part of the chamber were used as ports for injecting CH3Cl and extracting gas samples. A small ventilator in the lower part of the chamber ensured an even distribution of the gases in the chamber. B. Gas-tight plant chambers with both plants inside at the end of the incubation period. Aluminium foil was wrapped around the chambers during incubation with CH3OH to reduce photosynthesis and microbial CO2 consumption during the labelling experiment.

## Slide 2
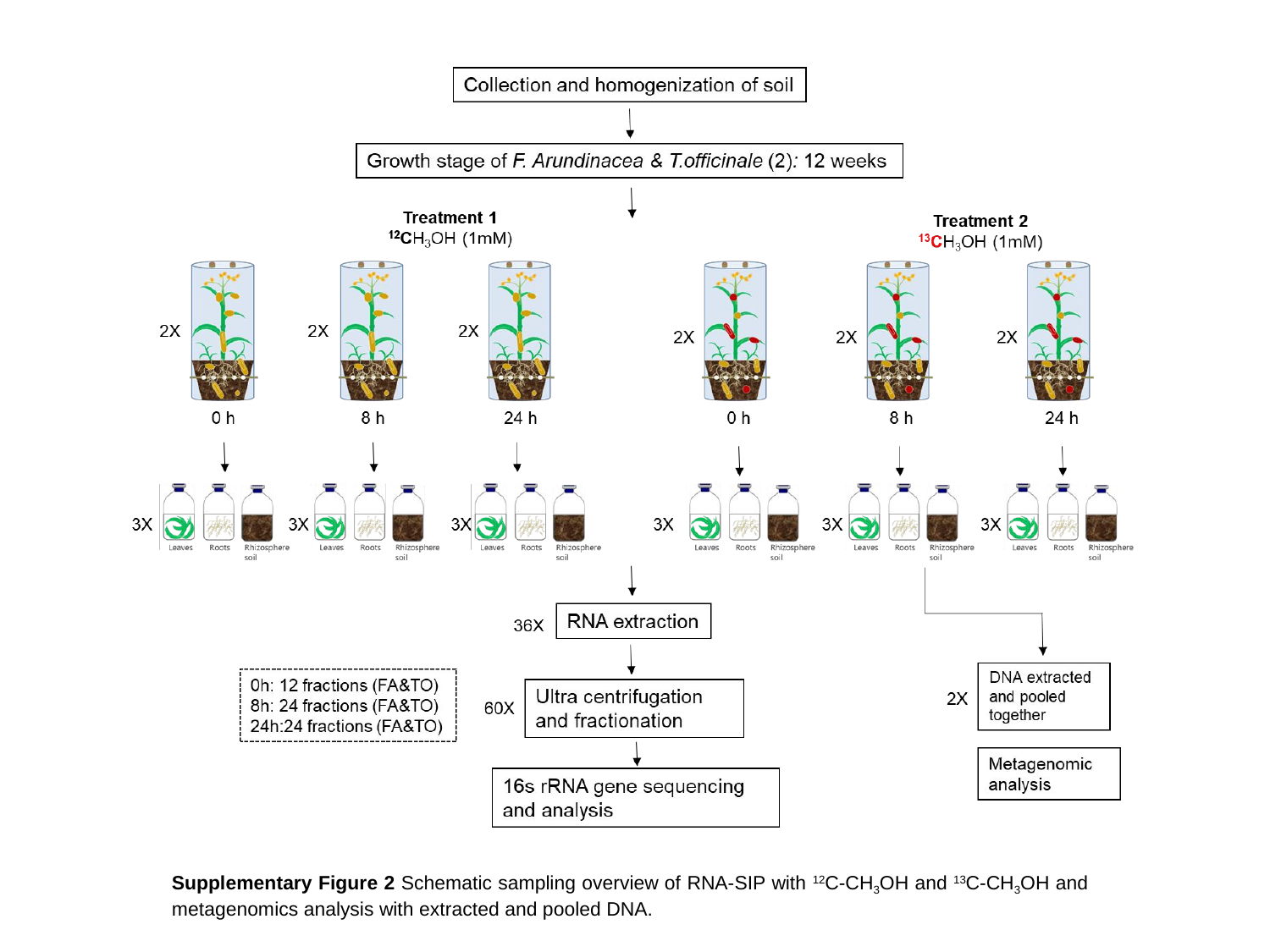

Supplementary Figure 2 Schematic sampling overview of RNA-SIP with 12C-CH3OH and 13C-CH3OH and metagenomics analysis with extracted and pooled DNA.

## Slide 3
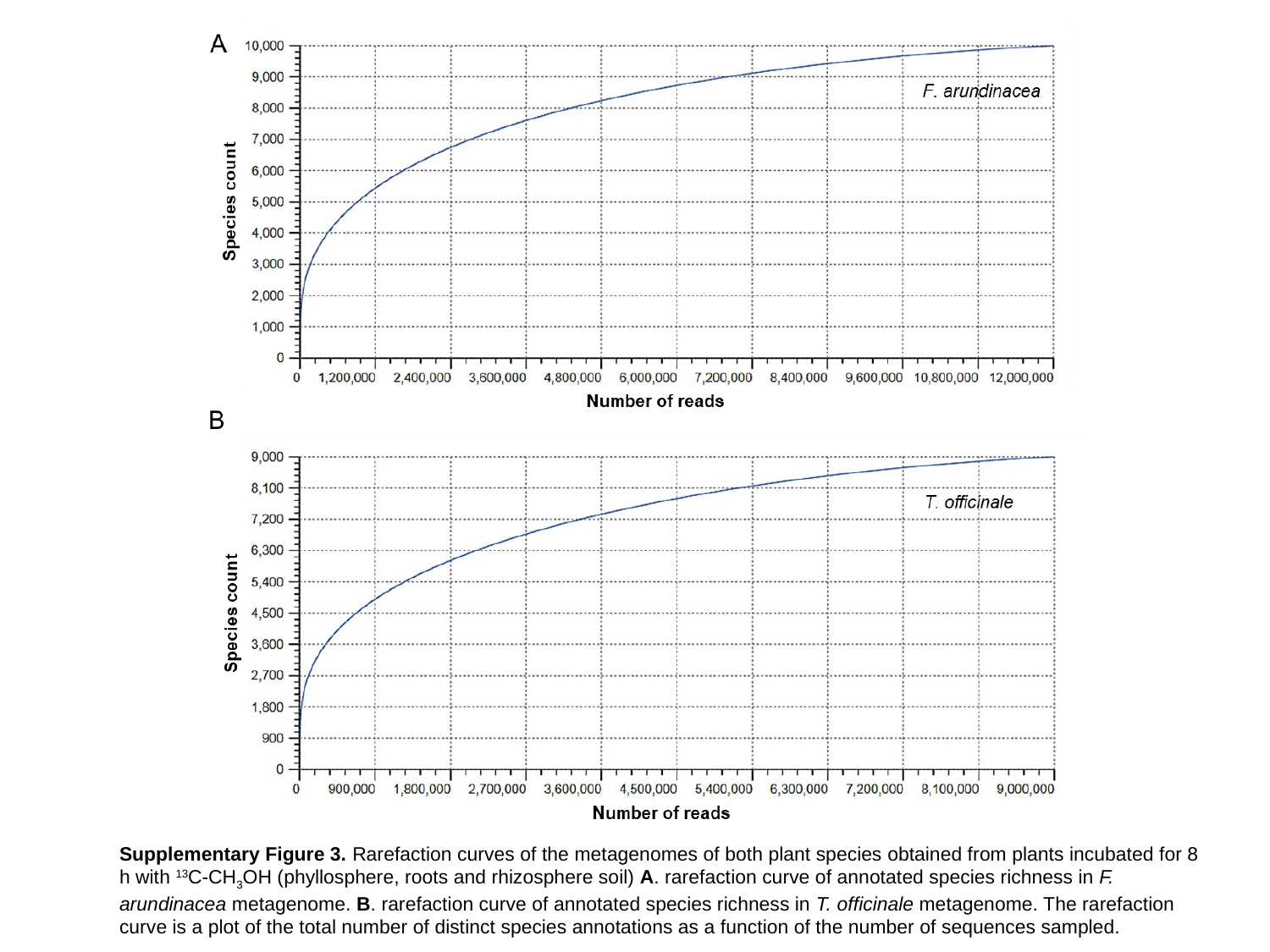

Supplementary Figure 3. Rarefaction curves of the metagenomes of both plant species obtained from plants incubated for 8 h with 13C-CH3OH (phyllosphere, roots and rhizosphere soil) A. rarefaction curve of annotated species richness in F. arundinacea metagenome. B. rarefaction curve of annotated species richness in T. officinale metagenome. The rarefaction curve is a plot of the total number of distinct species annotations as a function of the number of sequences sampled.

## Slide 4
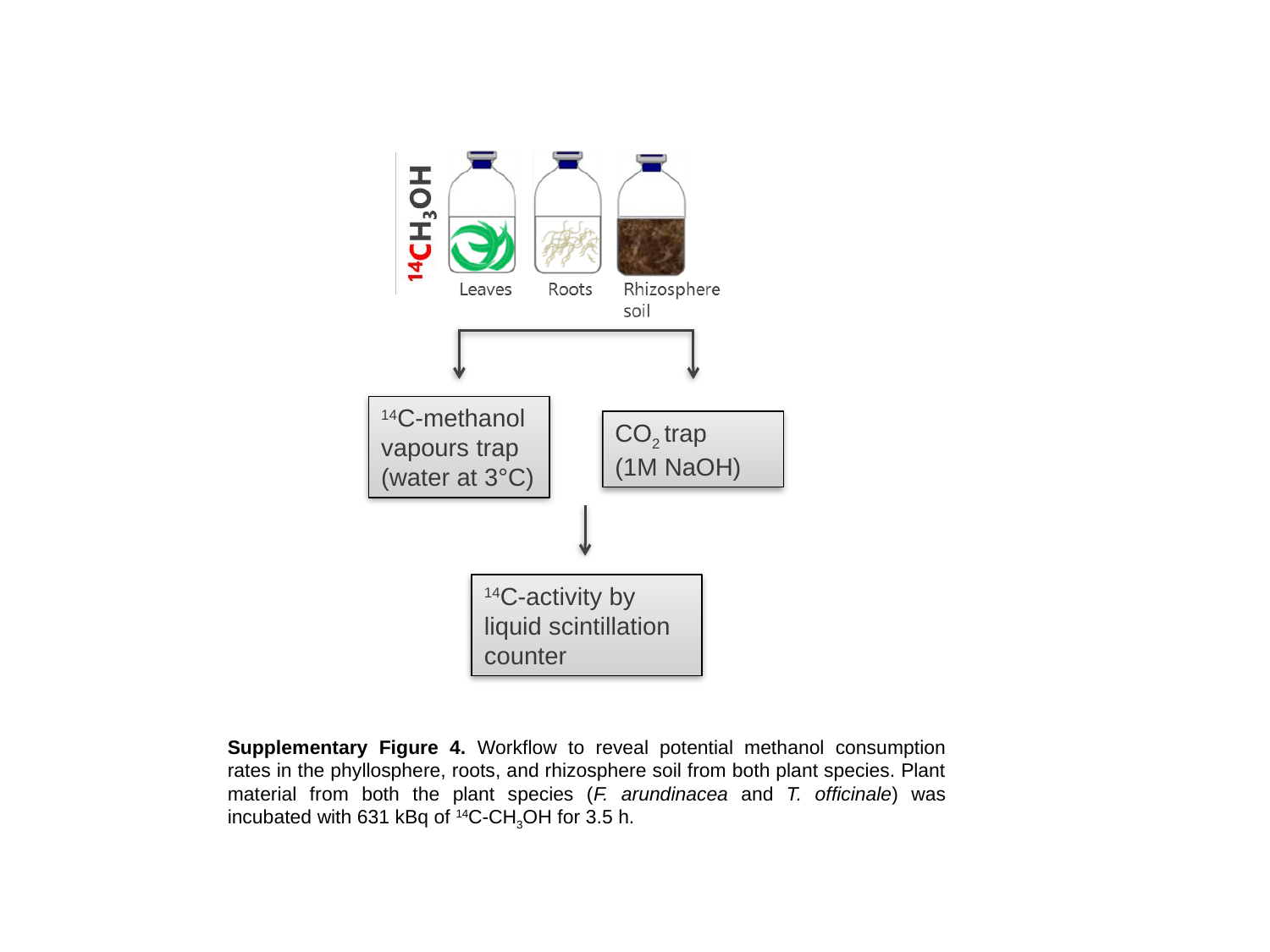

14C-methanol vapours trap
(water at 3°C)
CO2 trap
(1M NaOH)
14C-activity by liquid scintillation counter
Supplementary Figure 4. Workflow to reveal potential methanol consumption rates in the phyllosphere, roots, and rhizosphere soil from both plant species. Plant material from both the plant species (F. arundinacea and T. officinale) was incubated with 631 kBq of 14C-CH3OH for 3.5 h.

## Slide 5
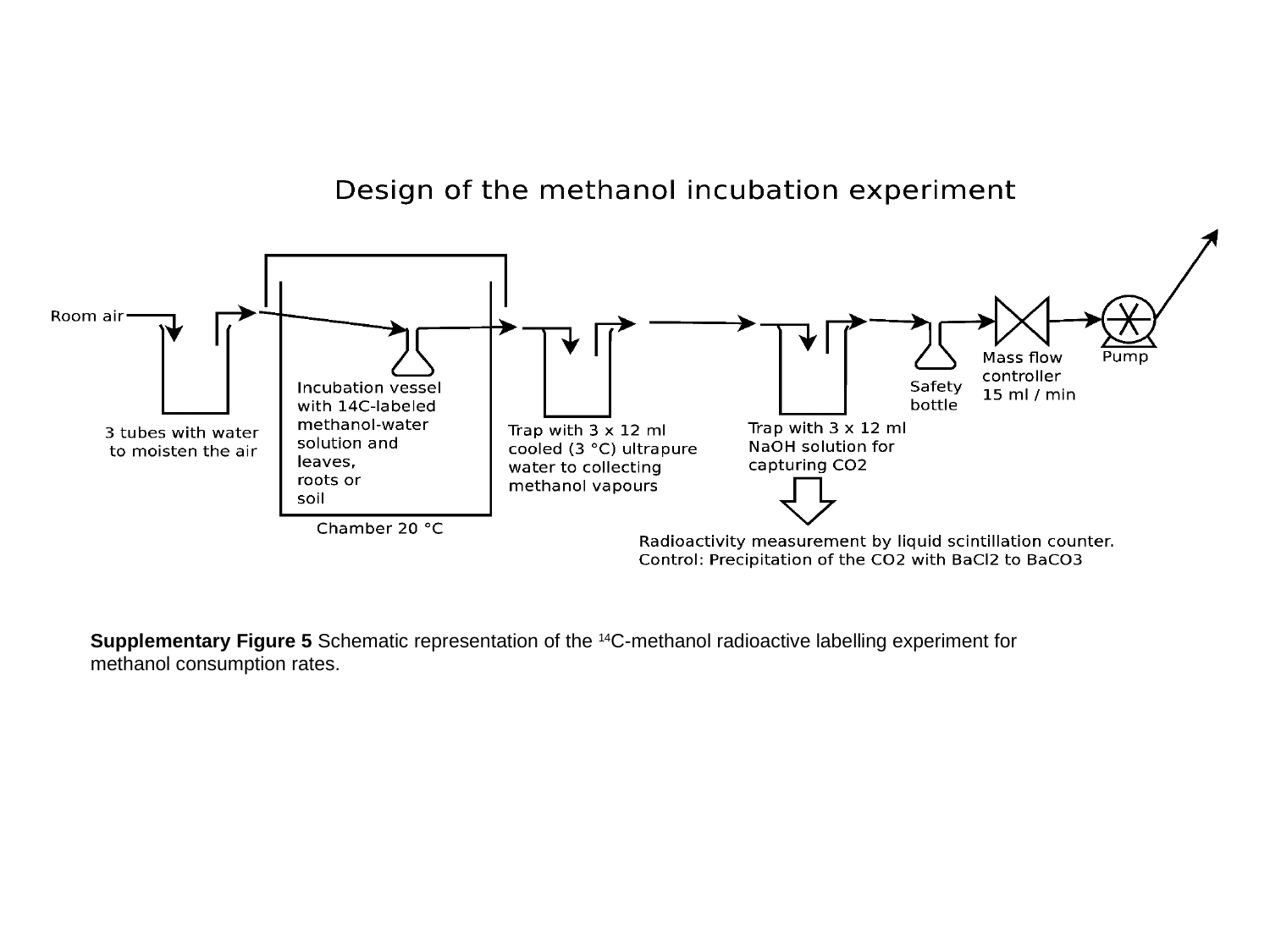

Supplementary Figure 5 Schematic representation of the 14C-methanol radioactive labelling experiment for methanol consumption rates.

## Slide 6
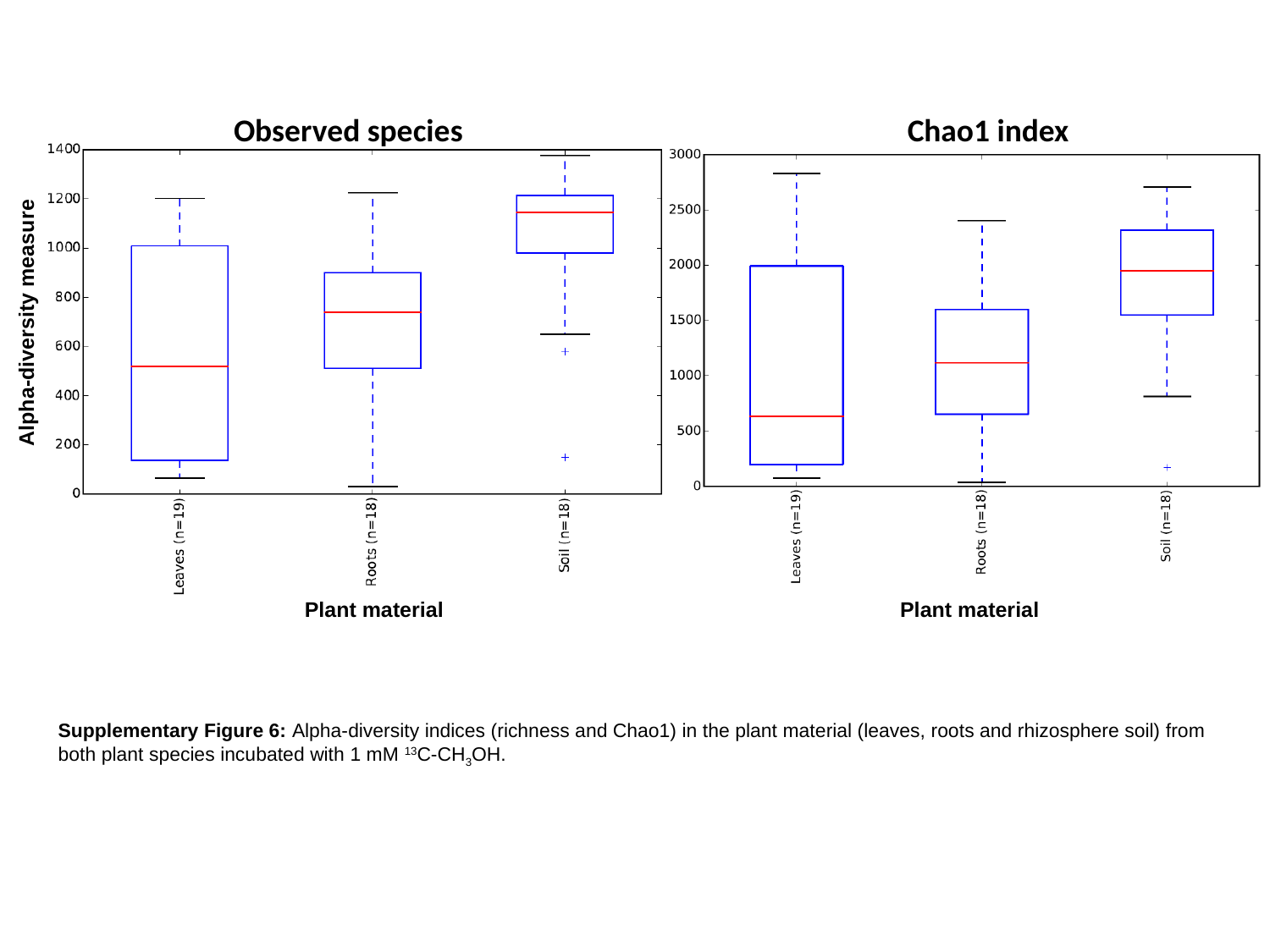

Observed species
Chao1 index
Alpha-diversity measure
Plant material
Plant material
Supplementary Figure 6: Alpha-diversity indices (richness and Chao1) in the plant material (leaves, roots and rhizosphere soil) from both plant species incubated with 1 mM 13C-CH3OH.

## Slide 7
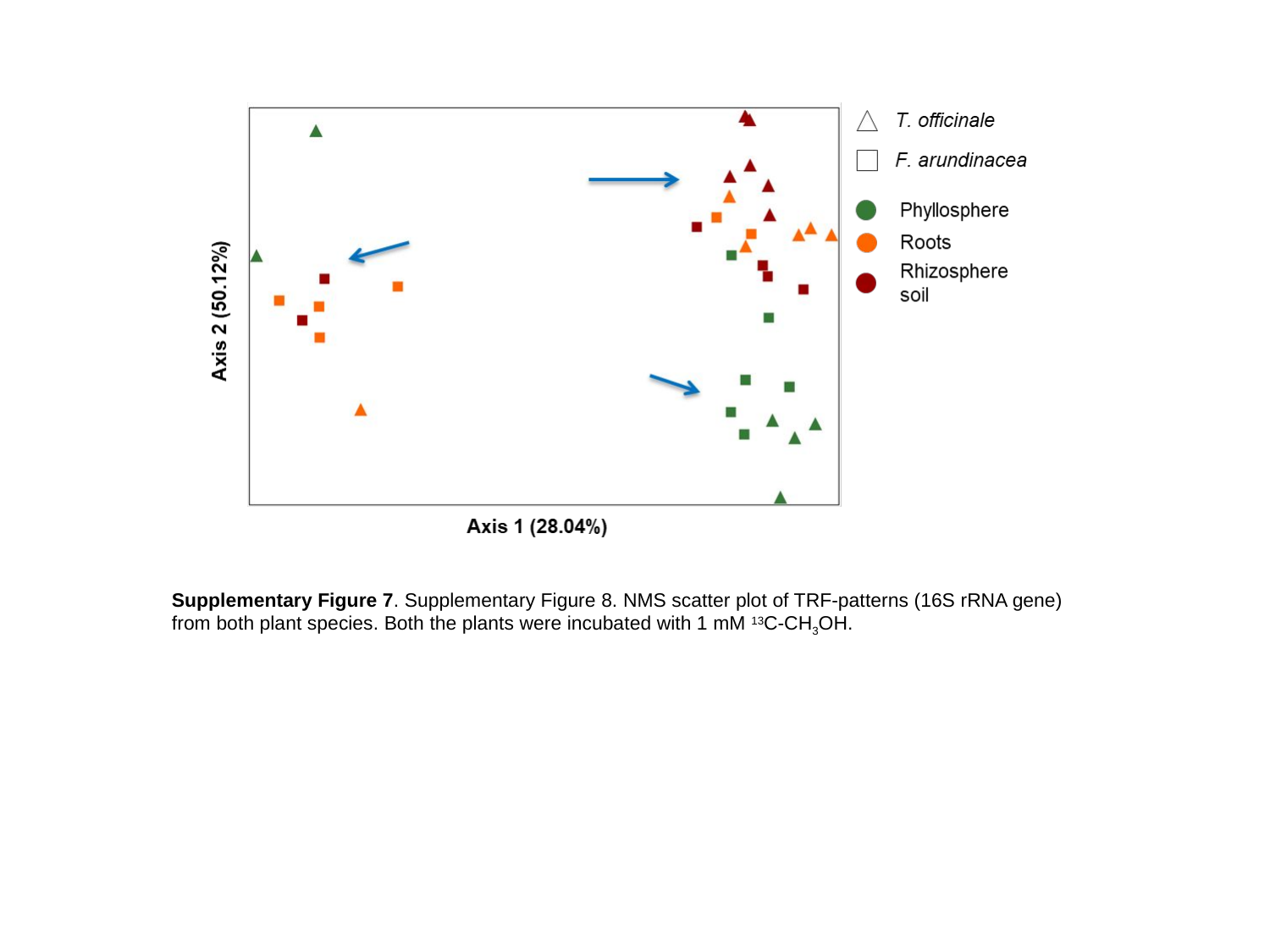

Supplementary Figure 7. Supplementary Figure 8. NMS scatter plot of TRF-patterns (16S rRNA gene) from both plant species. Both the plants were incubated with 1 mM 13C-CH3OH.

## Slide 8
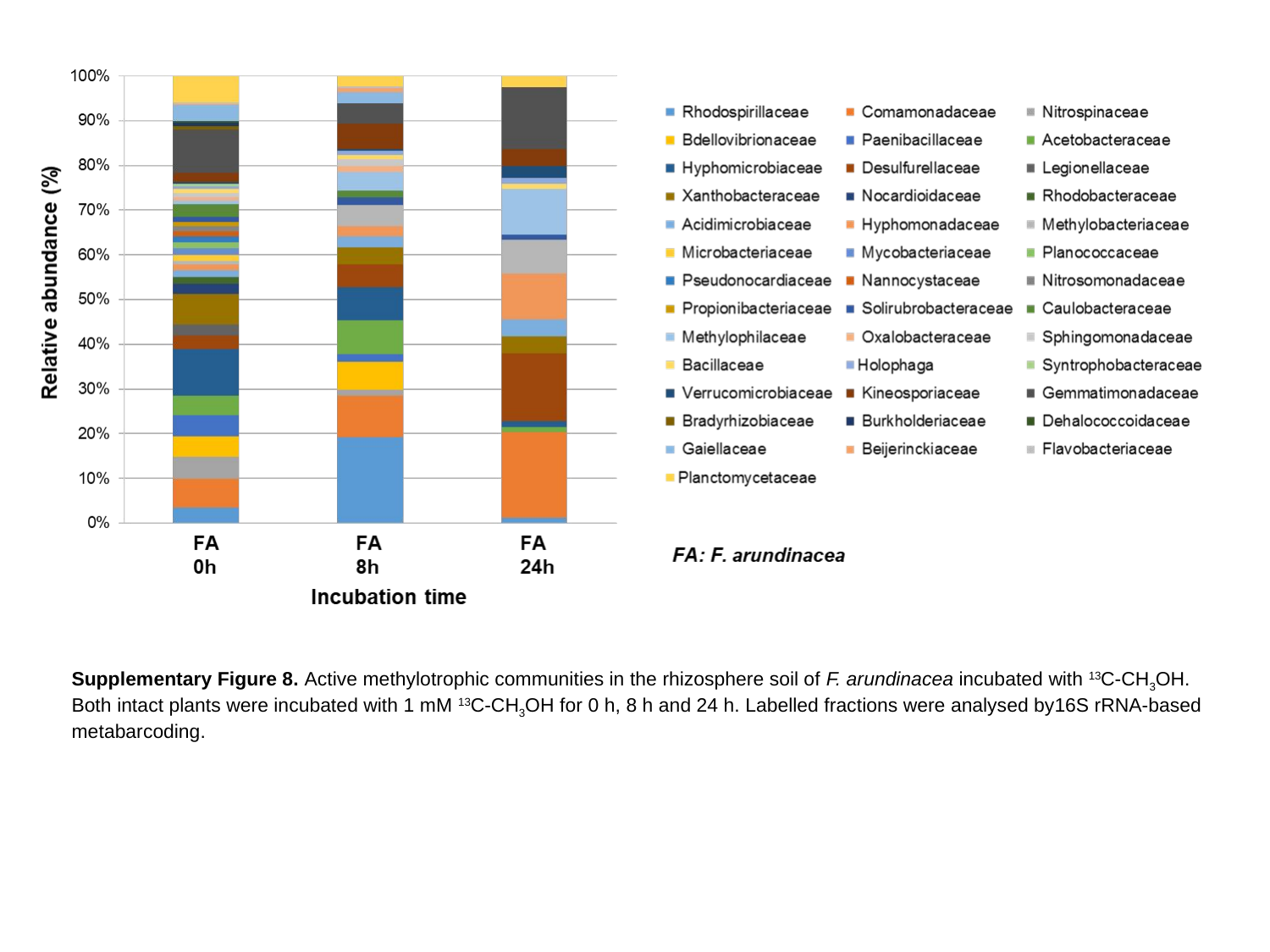

Supplementary Figure 8. Active methylotrophic communities in the rhizosphere soil of F. arundinacea incubated with 13C-CH3OH. Both intact plants were incubated with 1 mM 13C-CH3OH for 0 h, 8 h and 24 h. Labelled fractions were analysed by16S rRNA-based metabarcoding.

## Slide 9
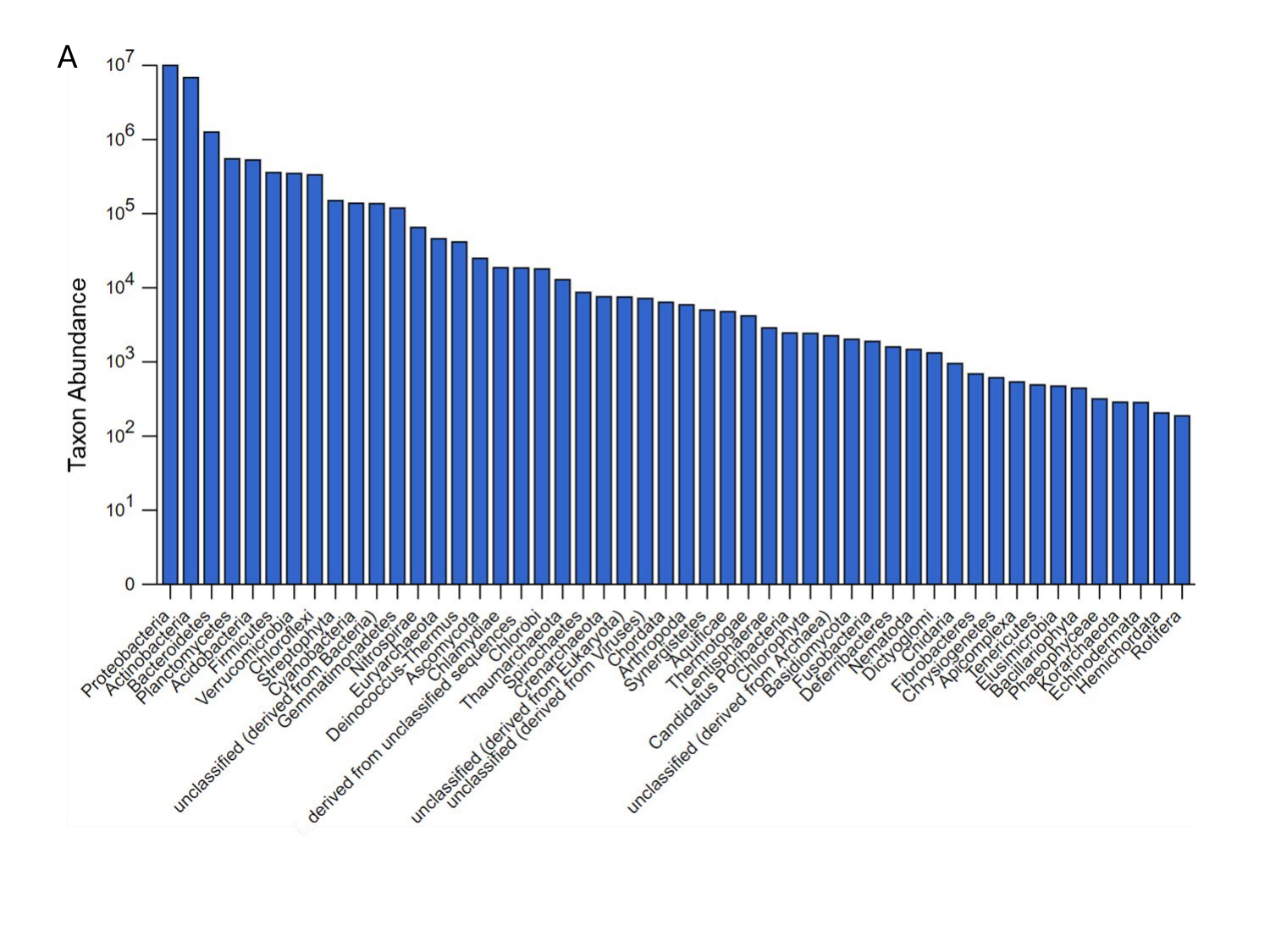

A

## Slide 10
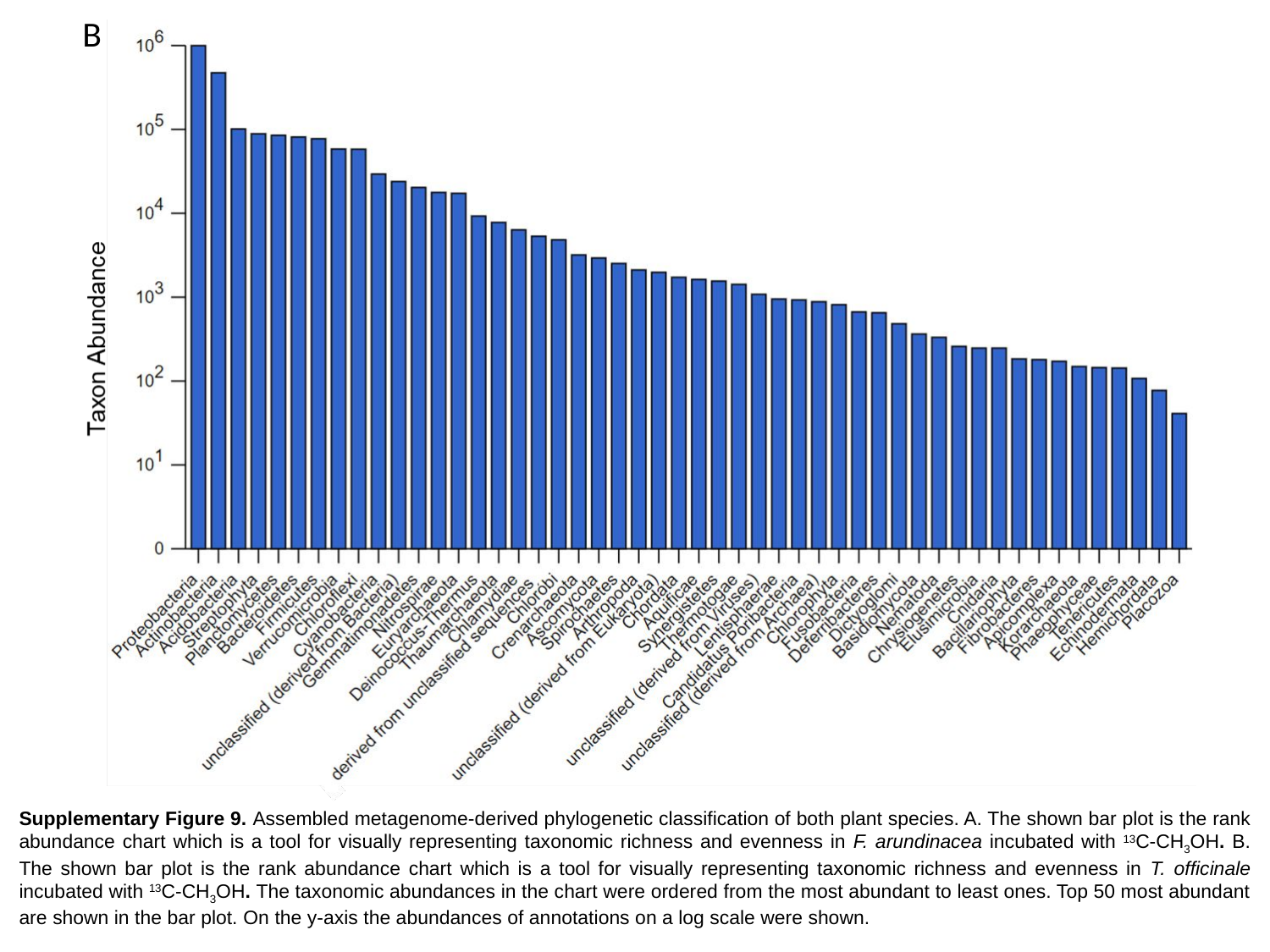

B
Supplementary Figure 9. Assembled metagenome-derived phylogenetic classification of both plant species. A. The shown bar plot is the rank abundance chart which is a tool for visually representing taxonomic richness and evenness in F. arundinacea incubated with 13C-CH3OH. B. The shown bar plot is the rank abundance chart which is a tool for visually representing taxonomic richness and evenness in T. officinale incubated with 13C-CH3OH. The taxonomic abundances in the chart were ordered from the most abundant to least ones. Top 50 most abundant are shown in the bar plot. On the y-axis the abundances of annotations on a log scale were shown.

## Slide 11
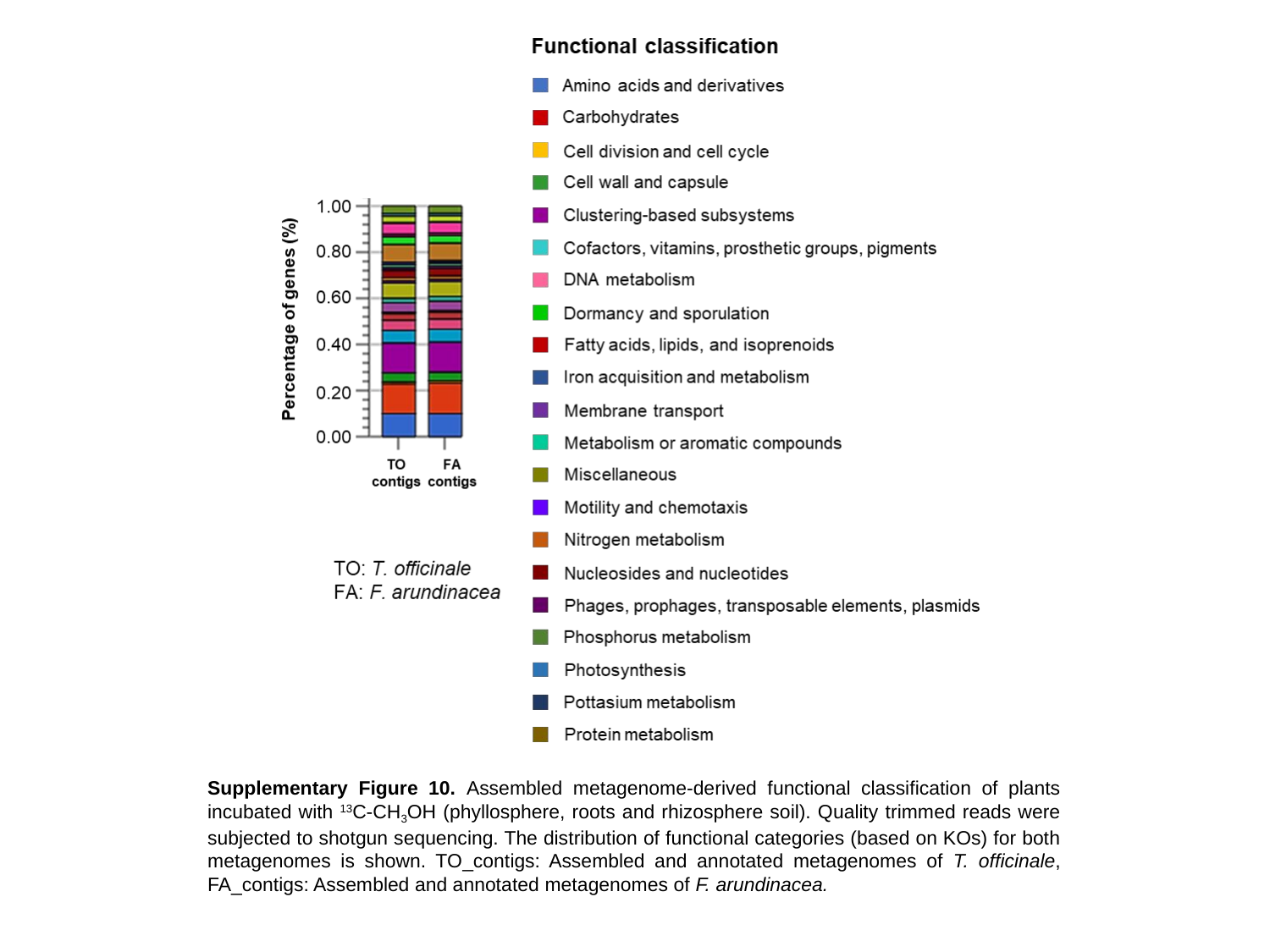

Supplementary Figure 10. Assembled metagenome-derived functional classification of plants incubated with 13C-CH3OH (phyllosphere, roots and rhizosphere soil). Quality trimmed reads were subjected to shotgun sequencing. The distribution of functional categories (based on KOs) for both metagenomes is shown. TO_contigs: Assembled and annotated metagenomes of T. officinale, FA_contigs: Assembled and annotated metagenomes of F. arundinacea.

## Slide 12
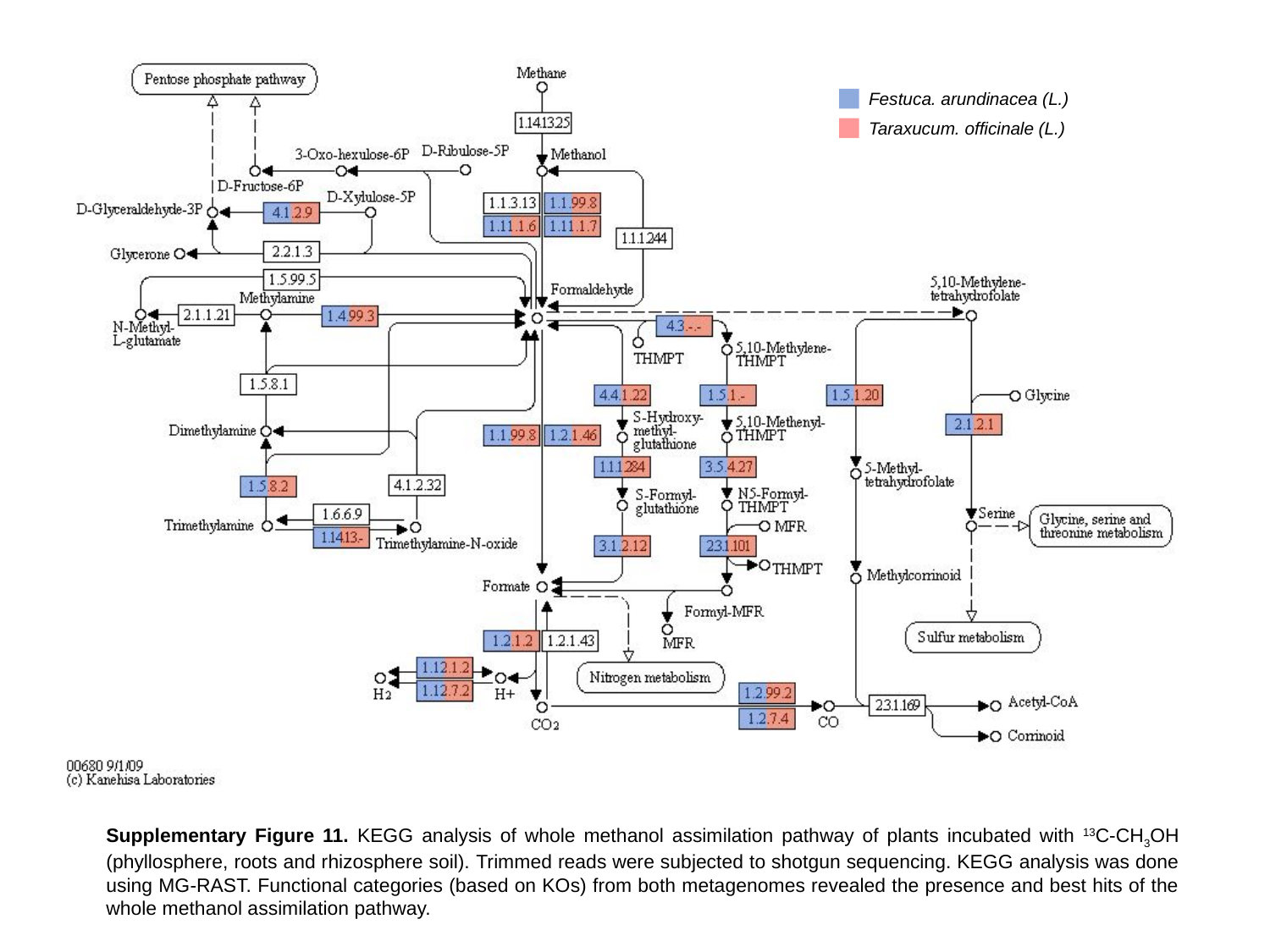

Festuca. arundinacea (L.)
Taraxucum. officinale (L.)
Supplementary Figure 11. KEGG analysis of whole methanol assimilation pathway of plants incubated with 13C-CH3OH (phyllosphere, roots and rhizosphere soil). Trimmed reads were subjected to shotgun sequencing. KEGG analysis was done using MG-RAST. Functional categories (based on KOs) from both metagenomes revealed the presence and best hits of the whole methanol assimilation pathway.

## Slide 13
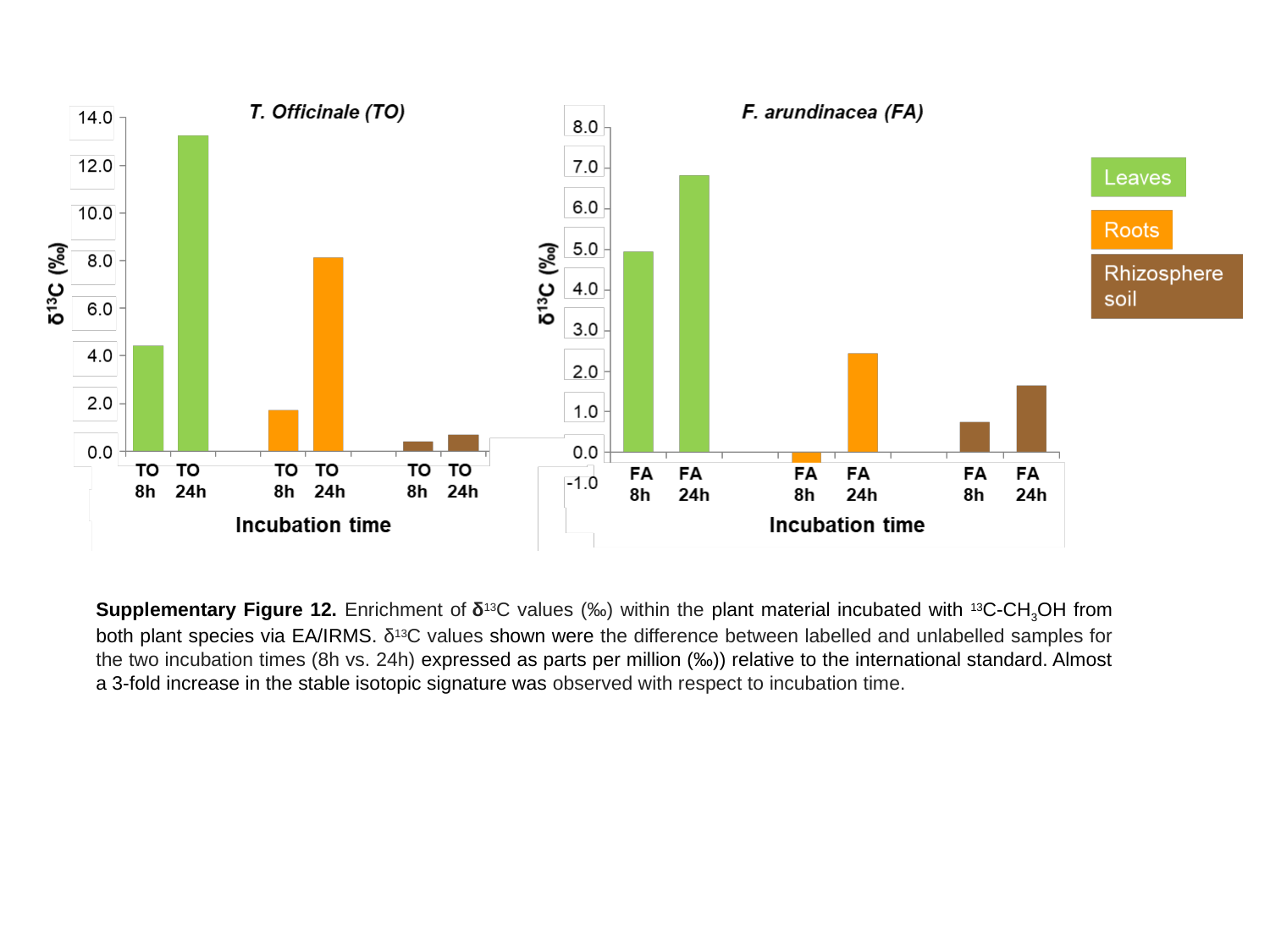

Supplementary Figure 12. Enrichment of δ13C values (‰) within the plant material incubated with 13C-CH3OH from both plant species via EA/IRMS. δ13C values shown were the difference between labelled and unlabelled samples for the two incubation times (8h vs. 24h) expressed as parts per million (‰)) relative to the international standard. Almost a 3-fold increase in the stable isotopic signature was observed with respect to incubation time.

## Slide 14
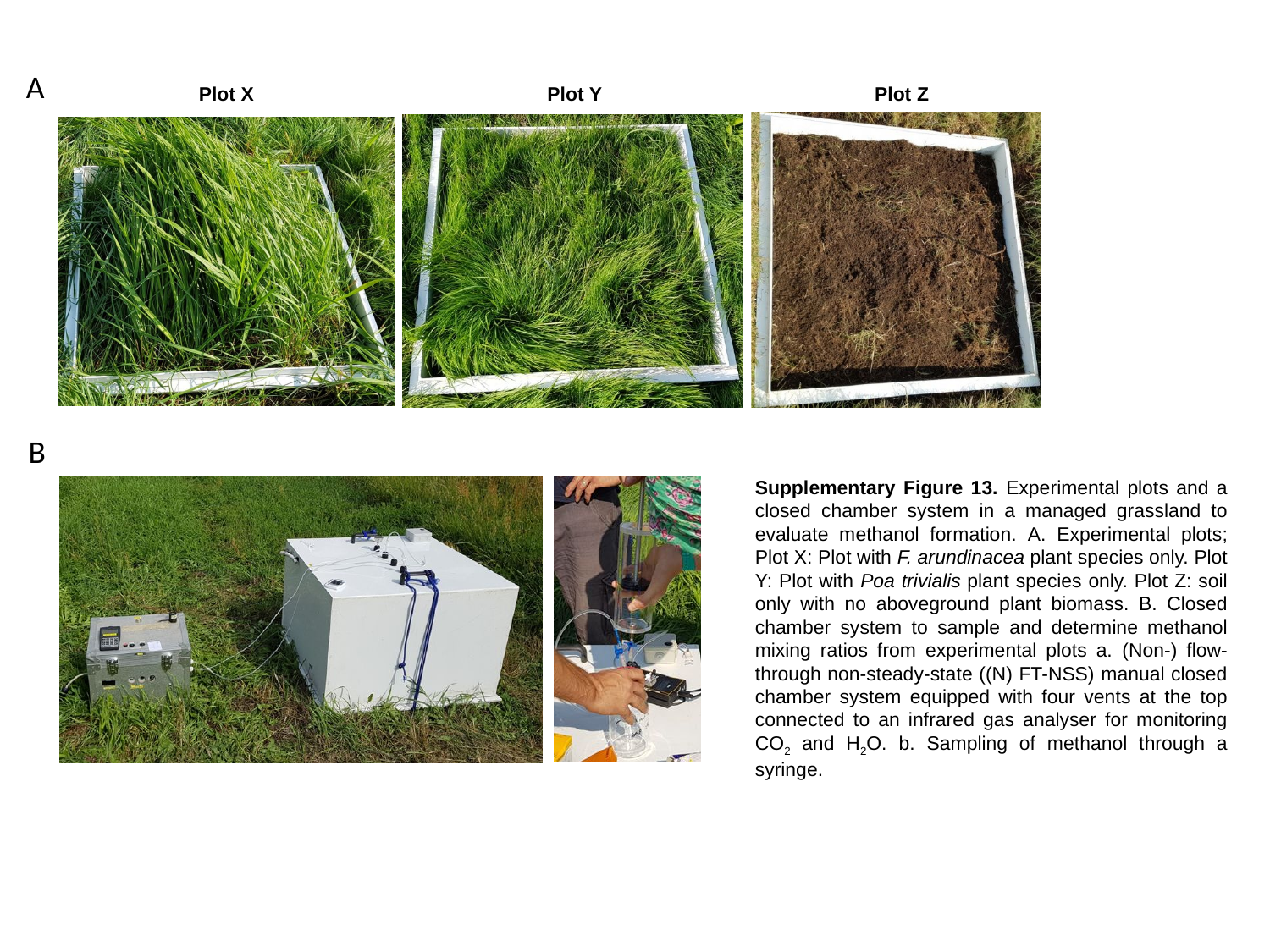

A
Plot X
Plot Y
Plot Z
B
Supplementary Figure 13. Experimental plots and a closed chamber system in a managed grassland to evaluate methanol formation. A. Experimental plots; Plot X: Plot with F. arundinacea plant species only. Plot Y: Plot with Poa trivialis plant species only. Plot Z: soil only with no aboveground plant biomass. B. Closed chamber system to sample and determine methanol mixing ratios from experimental plots a. (Non-) flow-through non-steady-state ((N) FT-NSS) manual closed chamber system equipped with four vents at the top connected to an infrared gas analyser for monitoring CO2 and H2O. b. Sampling of methanol through a syringe.

## Slide 15
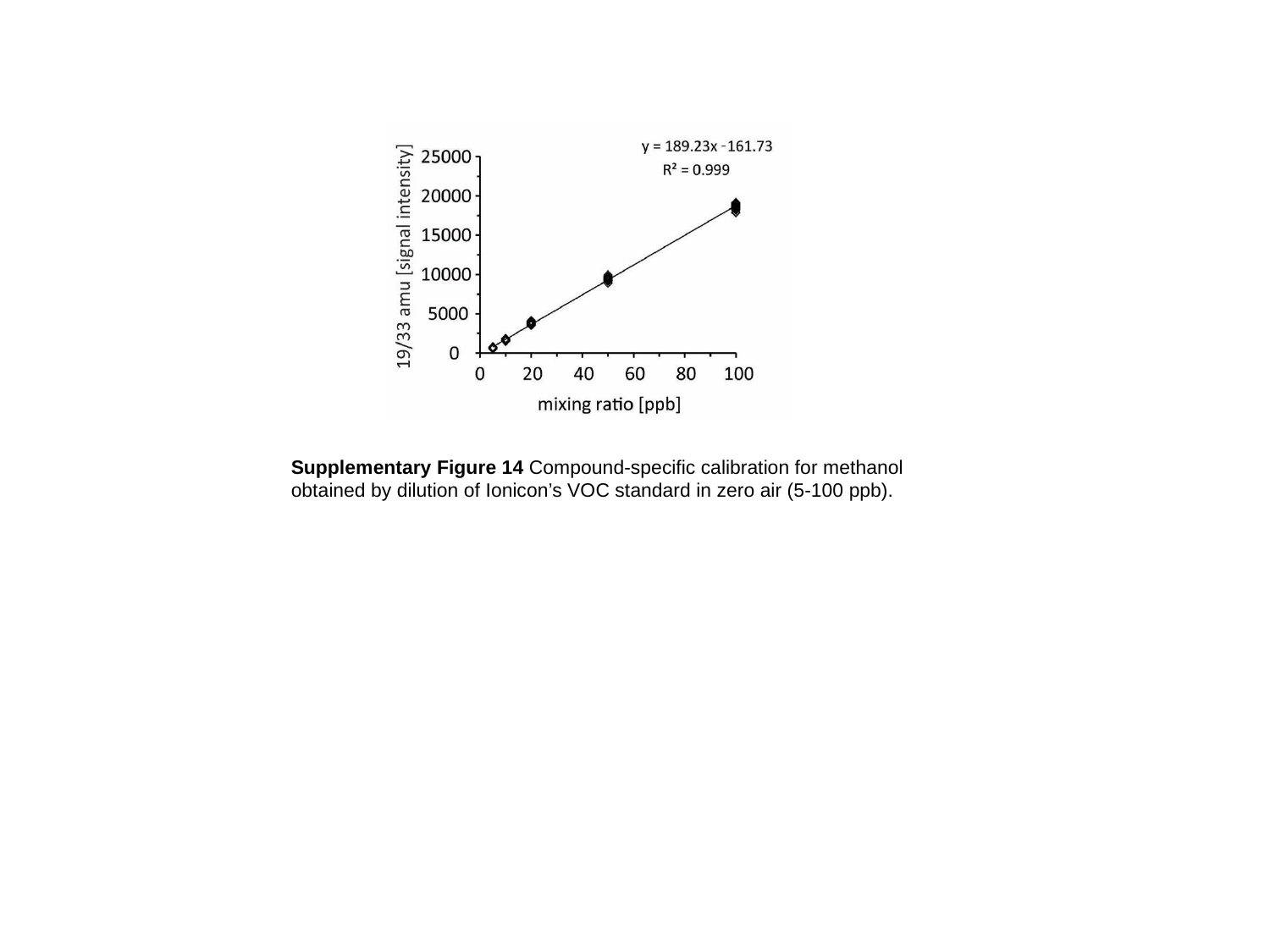

Supplementary Figure 14 Compound-specific calibration for methanol obtained by dilution of Ionicon’s VOC standard in zero air (5-100 ppb).
