# Supplementary material for: Methanol utilizers of the rhizosphere and phyllosphere of a common grass and forb host species
Source: Environ Microbiome. 2022 Jul 6;17:35. doi: 10.1186/s40793-022-00428-y (PMC9258066; doi:10.1186/s40793-022-00428-y)
Supplement: Supplementary file 2 — Additional file 2. Supplementary Table 1. [file 40793_2022_428_MOESM2_ESM.pdf]

Supplementary Table 1. δ^{13}C values (‰) of labelled and unlabelled samples for the two incubation times (8h vs. 24h)

		δ^{13}C values – unlabelled			δ^{13}C values - labelled		
Plant species	Time of incubation	Leaves	Roots	Soil	Leaves	Roots	Soil
T. officinale	8 h	-30.7	-28.1	-27.3	-26.3	-26.4	-26.9
T. officinale	24 h	-30.0	-27.0	-27.2	-16.8	-18.9	-26.5
F. arundinacea	8 h	-27.8	-26.8	-26.9	-22.9	-27.2	-26.1
F. arundinacea	24 h	-28.8	-27.5	-27.2	-22.0	-25.1	-25.6
