# Supplementary material for: Methanol utilizers of the rhizosphere and phyllosphere of a common grass and forb host species
Source: Environ Microbiome. 2022 Jul 6;17:35. doi: 10.1186/s40793-022-00428-y (PMC9258066; doi:10.1186/s40793-022-00428-y)
Supplement: Supplementary file 3 — Additional file 3. Supplementary information on the evaluation of methanol formation in a managed grassland. [file 40793_2022_428_MOESM3_ESM.pdf]

Supplementary information on the evaluation of methanol formation in a managed grassland

Site description and field experimental details

Spot check quantification of methanol formation was investigated in a managed grassland (see site details in the M&M section) with 3 different plots with 2 different grassland plants (*Festuca arundinacea* and *Poa trivialis*) and a plot with only soil, i.e., without aboveground plant biomass (Additional file 14: Supplementary figure 13). All three plots were equipped with two closed chambers (chamber A and chamber B) for collecting the samples to evaluate methanol concentrations (Additional file 14: Supplementary figure 13).Gas exchange was measured using a (non-)flow-through non-steady-state ((N)FT-NSS) manual closed chamber system (Livingston, G. P. and Hutchinson, 1995). The chamber (2 mm thick; V: 0.296 m^3; A: 0.5625 m^2 (0.75 m x 0.75 m)) used in this study was made up of white PVC and equipped with four vents at the top for connection: 1.) an infrared gas analyser (LI-850, LI-COR Biosciences, USA) for monitoring the development of CO_2 and H_2O concentrations during chamber deployment; 2.) a thermometer (DET1R, Voltcraft, Germany) to record the internal chamber air temperature during chamber deployment; 3.) pre-evacuated Duran pressure plus bottles (500 mL) with a two-way valve to obtain air samples during chamber deployment for subsequent methanol (VOC) analyses and a glass syringe (V: 500 mL) with approx. 100 mL of sample to create overpressure (approx. 1.2 bar) in the bottles (Additional file 14: Supplementary figure 13B). For measurements, the chamber was deployed on PVC frames (0.75 m x 0.75 m) inserted 5 cm deep into the soil. Chamber-frame airtight sealing was assured through rubber gaskets at the bottom of the chamber, a wind shield and rubber ropes for proper fixation of the chamber at the frame and controlled through CO_2 exchange measurements performed in parallel. Samples were taken every 0, 15, 30, 60, and 120 min after chamber closure in evacuated 500 mL glass bottles and were closed with rubber stoppers and a screw cap. Sampling was performed in triplicate (3 bottles from each time point and chamber).

Quantification of methanol formation in the managed grasslands using SIFT-MS

Mixing ratios for methanol from plots with 2 grassland plants, plots without aboveground biomass and in ambient air from the field site were quantified using selected-ion flow-tube mass spectrometry (SIFT-MS; SYFT technologies, Christchurch, New Zealand) in selected ion monitoring (SIM) mode with He 6.0 as the carrier gas (Westfalen, Münster, Germany). For measurements, the septum of the gastight bottles was pierced with a needle connected to 1/8 inch inert, black PFA tubing. Due to the slight overpressure in the gas bottles (approximately 1.2 bar), the gaseous sample was flushed into the instrument without any additional labour. Each sample was scanned for 60 seconds, equivalent to 12 data points from each scan. For calculations of mixing ratios, we did not use the first and last 3 data points of each scan and averaged the data collected from 15-42 seconds within each scan. SIFT-MS was calibrated by diluting Ionicon’s VOC standard (Ionicon, Innsbruck, Austria) in zero air obtained from a pure air generator (PAG 003, Ecophysics, Dürnten, Switzerland) in the range from 5-100 ppb (Additional file 17, Supplementary figure 14). Each mixing ratio was flushed through the system until the signal became stable and was then analysed for another 3 min before being set to the next higher mixing ratio.

To account for a potential loss in methanol between the time of sampling and analysis, we performed a recovery rate test for methanol by flushing bottles identical to those used in the field with Ionicon’s VOC standard diluted in zero air to a final mixing ratio of 10 ppb at a rate of 500 mL/min for 10 min. Mixing ratios of methanol were quantified immediately after flushing and again after 72 h. These measurements did not show any loss in methanol over a period of 3 days. As all samples taken in the field were analysed within 72 h, we assume that our results obtained in the laboratory are representative of the field fluxes of methanol from the studied grasslands.

A step towards methanol net surface emissions and their sources and sinks at the ecosystem level

Quantification of methanol formation using closed chambers and SIFT-MS in a managed grassland provided insights regarding sources and sinks within the plant holobiont. Methanol mixing ratios from three experimental plots with two different plant species showed higher methanol emissions than that from the plot without plant biomass (Figure. 7). The highest average mixing ratio was found in plot Y with *P. trivialis*, with a value of 28.2 ± 1.5 ppb methanol after 30 min of chamber closure. In plot X, with *F. arundinacea* species, the average methanol mixing ratio reached 25.1 ± 0.9 ppb after 30 min. The mixing ratio for methanol in soil plot Z did not exceed 14.5 ± 1.3 ppb and remained very stable over time. This might suggest a constant methanol background rather than in situ soil production of methanol over the 2 h period. Higher ambient air mixing ratios (17.1 ± 0.7 ppb) than those of the 'soil only' plots suggest that soil is not a net source of methanol but a sink. Higher methanol mixing ratios in plots with aboveground plant biomass can confirm that they are methanol sources

Figure legend

Additional file 1

Format: Additional_file_1.ppt

Supplementary Figure 14 Compound-specific calibration for methanol obtained by dilution of Ionicon's VOC standard in zero air (5-100 ppb).
