# Supplementary material for: Methanol utilizers of the rhizosphere and phyllosphere of a common grass and forb host species
Source: Environ Microbiome. 2022 Jul 6;17:35. doi: 10.1186/s40793-022-00428-y (PMC9258066; doi:10.1186/s40793-022-00428-y)
Supplement: Supplementary file 4 — Additional file 4. : Supplementary Table 2. [file 40793_2022_428_MOESM4_ESM.pdf]

Supplementary Table 2. Rate change in methanol mixing ratios of both plant species, soil and ambient air

**Sampling material**	**Rate (ppb/15 min)**	**Standard deviation**
Poa trivialis	0,488	0,237
F. arundinacea	0,446	0,192
Soil	0,013	0,074
Air	0	0
